# Structural Characterization and Molecular Dynamics Study of the REPI Fusion Protein from *Papaver somniferum* L.

**DOI:** 10.3390/biom14010002

**Published:** 2023-12-19

**Authors:** Alba Diaz-Bárcena, Luis Fernandez-Pacios, Patricia Giraldo

**Affiliations:** Department of Biotechnology-Plant Biology, School of Agricultural, Food and Biosystems Engineering, Universidad Politécnica de Madrid, 28040 Madrid, Spain; luis.fpacios@upm.es (L.F.-P.); patricia.giraldo@upm.es (P.G.)

**Keywords:** *Papaver somniferum*, REPI, STORR, protein structural modeling, molecular dynamics, electrostatic potential, substrate channeling

## Abstract

REPI is a pivotal point enzyme in plant benzylisoquinoline alkaloid metabolism as it promotes the evolution of the biosynthetic branch of morphinan alkaloids. Experimental studies of its activity led to the identification of two modules (DRS and DRR) that catalyze two sequential steps of the epimerization of (S)- to (R)-reticuline. Recently, special attention has been paid to its genetic characterization and evolutionary history, but no structural analyses of the REPI protein have been conducted to date. We present here a computational structural characterization of REPI with heme and NADP cofactors in the apo state and in three complexes with substrate (S)-reticuline in DRS and intermediate 1,2-dehydroreticuline in DRS and in DRR. Since no experimental structure exists for REPI, we used its AlphaFold model as a scaffold to build up these four systems, which were submitted to all-atom molecular dynamics (MD) simulations. A comparison of MD results for the four systems revealed key dynamic changes associated with cofactor and ligand binding and provided a dynamic picture of the evolution of their structures and interactions. We also explored the possible dynamic occurrence of tunnels and electrostatic highways potentially involved in alternative mechanisms for channeling the intermediate from DRS to DRR.

## 1. Introduction

Benzylisoquinoline alkaloids (BIAs) comprise over 2500 secondary metabolites mostly produced by plants of the genus Papaver [1,2]. Some BIA alkaloids present relevant pharmacological properties including the antitumoral effect of noscapine or the antimicrobial use of sanguinarine and berberine. However, the most notable application is the use of morphinan alkaloids as analgesics, both in direct use (codeine and morphine) and as a source for producing semi-synthetic derivatives (thebaine) [3,4,5]. Since the discovery of morphine in 1806, opioid-based analgesics have been essential for human civilizations and continue to be the most cost-effective treatment for severe pain and palliative care to date. Although different species of the Papaver genus accumulate a variety of BIAs, *Papaver somniferum* L. (opium poppy) is the only species that accumulates high levels of morphine and codeine for commercial production [6,7]. Great efforts have been made to replicate the morphinan biosynthetic pathway using metabolic engineering as microbial biomanufacturing platforms offer cheaper, expedited, and tightly controlled conditions compared to plant-based crop production [8,9,10]. However, the five chiral carbons of the morphinan alkaloid structure limit the efficiency of chemical synthesis, with no mechanism available to date to produce these compounds on a large scale [11]. The significance of the opium poppy as the sole source of morphinan opiates has established it as a model species for studying the genetics and biochemistry underlying BIA biosynthetic pathways [12,13,14]. The central intermediate in BIA metabolism is (S)-reticuline as it constitutes the branch point from which most structurally distinct subclasses of alkaloids are produced. This major branch gives rise to noscapine, sanguinarine and berberine alkaloids, beginning with the conversion of (S)-reticuline into (S)-scoulerine by the berberine bridge enzyme [1,2,14]. Alternatively, the epimerization of (S)-reticuline to (R)-reticuline opens the gateway to morphinan alkaloid biosynthesis. This key step consists of a first conversion of (S)-reticuline into an intermediate (1,2-dehydroreticuline), which is then reduced to (R)-reticuline. Initially, these reactions were thought to be catalyzed by two different enzymes. However, a fusion protein responsible for catalyzing both steps was discovered in 2015 and designated REPI (reticuline epimerase) or STORR ((S)- to (R)-reticuline) [5,10,15].

REPI is encoded by a 3 kb fusion gene which resulted from the duplication and subsequent deletion of two independent modules that were transcribed and translated together into a single 901-amino acid polypeptide [5,15]. Although paralogs of both modules have been described to exist independently in the genomes of most Papaver species, the REPI fusion gene has only been identified in those species that accumulate morphinan or promorphinan alkaloids [7,12]. In this way, the fusion event between the two independent domains seemed to be a key point in the morphine synthesis pathway evolution [5,7,15]. The N-terminal domain, called CYP82Y2 or DRS (1,2-dehydroreticuline synthase), is responsible for the conversion of (S)-reticuline into 1,2-dehydroreticuline and belongs to the cytochrome P450 family that uses heme as a cofactor. The C-terminal domain that catalyzes the conversion of 1,2-dehydroreticuline into (R)-reticuline using NADPH as cofactor is named DRR (1,2-dehydroreticuline reductase), and it belongs to the aldo-keto reductase (AKR) family. DRR is a cognate of the codeinone reductase (COR) enzyme, which acts downstream in the morphine synthesis pathway [15,16].

Although the main biosynthetic pathways leading to morphinan alkaloids have been elucidated and their enzymes have been functionally characterized, little is known about the three-dimensional (3D) structure of BIA proteins. So far, experimental structures exist for only four of the enzymes involved in morphinan alkaloid biosynthesis: SalR, THS, NISO, COR, and T6ODM [4,17,18,19,20]. These studies allowed for the identification of active sites and residues involved in binding distinct ligands. Indeed, the high sequence identity and phylogenetic proximity between the COR enzyme and the DRR module of REPI permitted a first homology model for DRR in 2021 once the crystal structure of COR was determined [19]. However, the sequence alignments of both proteins revealed many substitutions in DRR, including some of the residues that define the catalytic tetrad in COR [19]. The breakthrough advances in protein structure prediction represented by the development of AlphaFold2 [21] enabled the massive modeling of 3D structures with unparalleled reliability. In 2022, the latest release of the AlphaFold Protein Structure Database contained over 200 million structures [22] that provide broad coverage of UniProt. In particular, the 3D model structure of REPI (UniProt P0DKI7) included in the AlphaFold database was the starting point of the structural study presented here.

It has been suggested that the correlation between the REPI fusion gene and the ability to synthesize morphinan alkaloids is because this fusion promoted the efficient channeling of the intermediate, 1,2-dehydroreticuline [5,15]. The channeling of a substrate is a process in which a reaction intermediate is directly transferred from one active site to another, either in a multifunctional enzyme or in a multienzyme complex, without prior release into a bulk solvent [23,24,25,26]. Among the biological advantages associated with substrate channeling, the protection of unstable or reactive intermediates is of particular importance in plant secondary metabolism [27,28]. In REPI, substrate channeling could be advantageous in preventing the likely conversion of the unstable intermediate 1,2-dehydroreticuline into an enamine [15,29]. In fact, the study of microsomal fractions from Sacharomyces cerevisiae expressing the fusion REPI protein showed the conversion from (S)- to (R)- reticuline without the accumulation of 1,2-dehydroreticuline [5].

Although substrate channeling can play key roles in biological processes by enabling trafficking of intermediates that need to be shielded from the environment, the elucidation of structural details of ligand migration through tunnels or channels is particularly challenging [30]. In this regard, complex computational methods such as molecular dynamics (MD) simulations with further metadynamics calculations have been used to explore mechanisms of substrate channeling [31,32]. However, different easier-to-use methods have been specifically designed to identify and characterize tunnels and channels in proteins [33,34,35,36,37], thus providing useful information to address possible channeling processes without the extremely high computational cost of MD studies. These methods have been applied to proteins from the cytochrome P450 family as model candidates to analyze ligand migration and channeling. Given that their active sites are deeply buried, access/egress channels for P450 substrates are paramount [37,38,39,40].

On one hand, the study of the static structures of BIA enzymes helps us understand their interactions, identifying residues in binding sites, elucidating their physico-chemical properties (i.e., electrostatic potentials), and detecting possible tunnels or channels. On the other hand, the study of the dynamic evolution of these structures and all their features through MD simulations contributes to gaining detailed insight into their mechanisms of action. Both complementary structural studies are of key importance as they can be of invaluable help in the challenge to replicate morphinan biosynthesis through protein engineering [18,19,29]. However, except for the four cases mentioned [4,17,18,19,20], our structural picture of BIA enzymes is rather incomplete.

Aiming to contribute to expanding our current knowledge of BIA enzymes, we present in this work a structural characterization of REPI using its AlphaFold model structure as an initial scaffold to build models of its complexes with cofactors, substrate (S)-reticuline, and intermediate 1,2-dehydroreticuline which were then submitted to all-atom 400 ns MD simulations. We studied the dynamic evolution of (a) apo-REPI (no ligands), (b) REPI with (S)-reticuline at DRS, (c) REPI with 1,2-dehydroreticuline at DRS, and (d) REPI with 1,2-dehydroreticuline at DRR. By comparing the MD results of these four systems, we were able to identify key dynamic changes associated with different ligand bindings as well as the evolution of their structures, interaction energies, and other features. We also explored in the MD simulations the possible occurrence of tunnels or electrostatic highways potentially involved in alternative mechanisms for channeling the intermediate 1,2-dehydroreticuline from the DRS heme site to the DRR NADPH active site. Even though computational analyses have a certain associated uncertainty, the reliable MD-based methodology used in this work and the high confidence of the initial AlphaFold model taken as an initial scaffold for constructing the different REPI systems permit us to make predictions to be further tested in the laboratory.

## 2. Materials and Methods

### 2.1. Structures of Proteins, Cofactors, and Ligands

**Figure 1:**
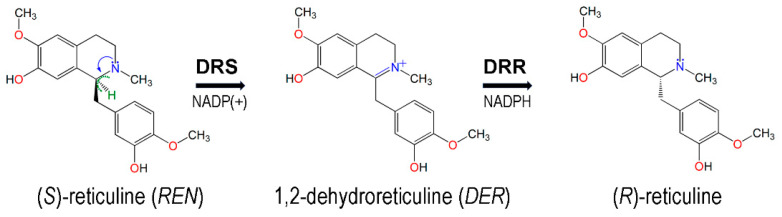
Epimerization of (S)-reticuline to (R)-reticuline catalyzed by REPI. The DRS module oxidizes (S)-reticuline (abbreviated as REN throughout this article) by removing the hydride marked in green and its bond electron pair. This results in C=N double-bond formation with the lone electron pair from N that thus becomes positively charged. The intermediate, 1,2-dehydroticuline (abbreviated as DER throughout this article), is reduced by the DRR module by adding a hydride atom that restores the C-N bond in the (R) enantiomer.

The 3D model structure of REPI was downloaded from the AlphaFold Protein Structure Database (https://alphafold.ebi.ac.uk/ (first accessed on 15 December 2022 and then confirmed on 4 December 2023)) [22], using the entry corresponding to the bifunctional protein of the *STORR* gene from *Papaver somniferum* id. P0DKI7 [5] in UniProt (https://www.uniprot.org/ (accessed on 15 December 2022)) [41]. The REPI model is composed of two well-differentiated modules, DRS and DRR. DRS first oxidizes (S)-reticuline to the quaternary positively charged iminium cationic intermediate 1,2-dehydroreticuline, which is then reduced by DRR to (R)-reticuline (Figure 1).

Similar structures in the Protein Data Bank (https://www.rcsb.org/ (accessed on 5 June 2023)) [42] were searched for DRS and DRR domains with Dali (http://ekhidna2.biocenter.helsinki.fi/dali/ (accessed on 5 June 2023)) [43]. Structural similarity between these domains and proteins with the highest Dali Z-scores was measured using the TM-score provided by TM-align (https://zhanggroup.org/TM-align/ (accesed on 7 June 2023)) [44]. This score scales the similarity between two proteins in a range (0, 1), where 1 means a perfect match, TM-score > 0.5 suggests the same fold, and values smaller than 0.2 indicate unrelated proteins.

Since AlphaFold structures have neither cofactors nor ligands, heme and NADP(+) or NADPH were first inserted in the REPI modules as follows. The protein with the highest Dali Z-score for comparison with DRS was the oxidoreductase CYP76AH3 from *Salvia miltiorrhiza* in a crystal structure (PDB id. 7X2Q [45]) that includes heme. An initial geometry for heme inserted into DRS was obtained upon superimposing DRS and 7X2Q and then adding the coordinates of the heme from 7X2Q to DRS coordinates. This superposition provided an excellent structural alignment (RMSD = 1.19 Å, with 329/456 residues aligned with the MatchMaker tool of Chimera 1.17 [46]) with the spatial positions of the cysteines that coordinate heme iron (C513 in DRS; C437 in 7X2Q) nearly coincident. In this initial geometry of the DRS-heme complex, the distance between C513 sulfur and heme iron is 2.34 Å, a value in close agreement with that observed in Extended X-ray Absorption Fine Structure (EXAFS) measurements in cytochrome P450s [47]. In the comparison for DRR, the protein with the highest Dali Z-score was apo-COR in a crystal structure (PDB id. 7MBF [19]) with no cofactor. Because of this, we used the protein with the second highest Dali Z-score, AKR4C17 from *Echinochloa colona* in a crystal structure, for its complex with NADP(+) (PDB id. 7F7K [48]). The same procedure used for heme was applied here, again obtaining a structural alignment (RMSD = 0.89 Å with 275/309 residues aligned) good enough to achieve a reliable initial geometry for the DRR-NADP(+) complex. The initial geometry for the DRR-NADPH complex was obtained by just modifying the proper N and C atoms of the nicotinamide ring to convert NADP(+) into NADPH, using the Build Structure tool in Chimera 1.17. These initial geometries of DRS-heme, the DRR-NADP(+), and DRR-NADPH complexes, were then optimized in water and 0.150 M of NaCl at 20,000 conjugate gradient steps following the minimization protocol indicated in Section 2.3 that includes the addition of hydrogens absent in the crystal structures used.

The 3D structure of (S)-reticuline, the substrate of DRS-heme, was taken from the crystal structure of the berberine bridge enzyme (BBE) from the California poppy *Eschscholzia californica* in complex with (S)-reticuline (PDB id. 3D2D [49]), named “REN” in this PDB entry, a symbol that will be used herein to abbreviate this substrate. The BBE catalyzes the conversion of (S)-reticuline into (S)-scoulerine and is the only protein with an experimental structure in the PDB that has REN as a ligand (which is also present in two variants of the BBE, PDB entries 3FWA and 4EC3). The intermediate of REPI, 1,2-dehydroreticuline (hereafter abbreviated as “DER”), the product of DRS and the substrate of DRR, is not present in any structure in the PDB. Therefore, as initial geometry for DER, we downloaded from PubChem (https://pubchem.ncbi.nlm.nih.gov/ (accessed on 16 January 2023)) [50] the 3D model of conformer 1 of DER in this database (compound CID 440930, formula C_19_H_22_NO_4_^+^).

### 2.2. Structures of Protein–Cofactor–Ligand Complexes

We present an MD study of four REPI systems: (a) REPI cofactors without a ligand, (b) REPI with REN at the heme site of DRS, (c) REPI with DER at the heme site of DRS, and (d) REPI with DER at the NADPH site of DRR. System (a) is composed of both DRS-heme and DRR-NADP(+) complexes; hence, its initial structure was just the optimized geometries of separate DRS-heme and DRR-NADP(+) mounted together on the AlphaFold initial model which was used as a scaffold. Systems (b) and (c) involved placing REN or DER at the heme site of DRS with NADP(+) in DRR in both cases. System (d) involved placing DER at the NADPH site of DRR. We resorted to docking calculations to obtain initial geometries of REN or DER at their corresponding cofactor sites using AutoDock Vina 1.2.3 [51]. A molecule of REN or DER with the geometries indicated in Section 2.1 was the “ligand”, while a DRS-heme or DRR-NADPH complex, with their optimized geometries obtained as explained in Section 2.1, was the “receptor”. A search was performed in blind-docking mode, that is, using an unrestricted search space around the whole receptor. A maximum of ten poses were requested, and three rounds of docking calculations were performed in all cases with the program vina-1.2.3 [52]. After checking for consistency in all the poses, we selected the best solution as that with the lowest predicted binding affinity, D*G*. These best solutions corresponded to D*G* values (kcal/mol) of −8.08 for DRS-heme-REN and −8.99 for DRR-NADPH-DER. Since DER is the intermediate formed after the catalytic activity of DRS-heme, the initial geometry for system (c) was obtained by just editing the REN molecule in the best docking solution for system (b) to convert it into DER by removing the H atom (colored green in Figure 1) in the -CH-N- single bond to obtain the -C=N^+^- double bond, a change carried out using the Build Structure tool of Chimera 1.17.

### 2.3. All-Atom Molecular Dynamics (MD) Calculations

The geometries of complexes (a)–(d) were then optimized to obtain the initial structures for all-atom MD calculations. To this end, all complexes were parametrized for the CHARMM 3.6 force field [53] with the c36md parameter set for proteins [54] using the PDB Reader service of the CHARMM-GUI server (https://charmm-gui.org/ (accessed on 18 January 2023)) [55,56]. Residue C513 of DRS was parametrized to be linked to heme iron (CHARMM patch “CYM” for cysteines). Periodic solvation boxes were then constructed with 14 Å margins by adding water molecules according to the TIP3P model of liquid water [57] and Na^+^ and Cl^−^ ions to counter the total electric charges of the systems while setting a 0.150 M salt concentration. The CHARMM atomic charges of all atoms were added, checking that the total charges of the proteins, cofactors, ligands, and ions were properly reproduced. These settings gave rise to the following numbers of atoms: 164,682, 164,711, 164,706, and 164,894 for systems (a)–(d), respectively. Initial geometries were optimized at 20,000 conjugate gradient minimization steps with the high-performance computing (HPC) power MPI version of NAMD 2.14 [58] to obtain initial structures for MD simulations that were then equilibrated as follows: (1) Systems were heated from 0 to 100 K in the canonical (NVT) ensemble with all atoms except those of water molecules subjected to a 5 kcal mol^−1^ Å^−2^ harmonic potential constraint for 1000 calculation steps. (2) After resetting velocities at 100 K, the temperature was raised from 100 to 300 K for 25,000 steps in the NVT ensemble with same constraints as in (1). (3) After resetting velocities at 300 K, the systems were equilibrated in the isothermal–isobaric (NPT) ensemble at P = 1 atm and T = 300 K with all atoms except those of the water molecules subjected to a 1 kcal mol^−1^ Å^−2^ harmonic potential constraint for 500,000 calculation steps. (4) The systems were then re-equilibrated at 300 K in the NPT ensemble for 1,500,000 calculation steps without any constraint to relax all atoms. All equilibration steps were performed at 2 fs time steps. The four systems (a)–(d) were found to be completely equilibrated upon analyzing the full trajectories of (1)–(4) and checking that the P, T, V, total energy, and RMSDs of the backbone atoms showed totally stabilized patterns.

At this point, every system was ready for simulation production runs performed in the NPT ensemble at 2 fs time steps for 400 ns, thus consisting of 200 million calculation steps. Both equilibration and simulation runs used the SHAKE algorithm [59], a 10 Å cutoff applied to short-range non-bonded interactions, and the particle-mesh Ewald summation method [60] for long-range electrostatics. Langevin dynamics for a constant T = 300 K control and the Nosé–Hoover Langevin piston method for a constant P = 1 atm control [61] were used. MD calculations were performed using the HPC Power MPI version of NAMD 2.14, using the Magerit3 supercomputer of Universidad Politécnica de Madrid (https://www.cesvima.upm.es/). Using 160 processors (4 nodes) in this HPC supercomputing facility, wall-clock times were 678.4, 680.1, 730.1, and 643.4 h for systems (a)–(d), respectively. The MD production run calculations thus amounted to a total elapsed time of 2732 h, i.e., 113.8 days. NAMD output was stored every 50,000 steps to produce trajectories composed of 4000 frames that were processed and analyzed using VMD 1.9.3 [62]. Several Tcl/Tk scripts for VMD and in-house programs were written to obtain from those trajectories the variations in the different properties presented and discussed in this work.

### 2.4. Structural Alignment, Assignment of Secondary Structure, Detection of Channels, and Molecular Graphics

Structural alignments were obtained using two different procedures: the *MatchMaker* tool [63] of Chimera 1.17 and the Combinatorial Extension (CE) algorithm [64] implemented in the command cealign of PyMOL 2.5.5 [65]. A secondary structure in protein 3D-coordinate *pdb* files was assigned with DSSP [66], using dssp 3.0.0 (https://github.com/PDB-REDO/dssp). A secondary structure along MD trajectories was obtained with the Timeline tool of VMD 1.9.3, and in-house routines were used to process its output for preparing heatmap plots.

The MOLE 2.5 program (https://webchem.ncbr.muni.cz/Platform/App/Mole) [37] was used for channel identification and characterization. This software allows for the rapid detection and physico-chemical characterization of tunnels, pores, and cavities in proteins. It implements the algorithm MOLE 2.0, which uses a Voronoi diagram based on the Delaunay triangulation of atomic centers to approximate the molecular surface, and a method to identify possible start and end points of channels using Delaunay tetrahedrons [35,37]. Potential start and end points can be specified in an automatic (“computed”) way if defined by the centers of the deepest tetrahedrons in each cavity or in a user-defined way if specific 3D points are given as an input. Channels are then computed as the shortest paths between all pairs of start and end points in the same cavity. Since this procedure usually generates a great number of candidates, the selection of the most relevant channels is finally made by using tunable parameters that control the maximum length, minimum radius, etc. [37,38]. After exploring options to reproduce benchmark data [38], we used the following settings (Å): Probe Radius 5, Interior Threshold 1.5, Cutoff Ratio 0.5, and default values for Minimum Depth, Bottleneck Length, Bottleneck Radius, Origin Radius, and Surface Cover Radius. Additionally, only channels longer than 15 Å were considered relevant [38] in REPI. The trajectories for the four systems (a)–(d) were scanned for channel opening events, and the selected frames were subjected to an analysis with MOLE 2.5. In these cases, together with the automatic “computed” start points identified by the MOLE 2.0 algorithm, we defined three additional start points at heme, at NADP, and at both heme and NADP simultaneously (Section 3.6). Results were exported to PyMOL scripts for graphical representation. Molecular graphics were prepared and rendered with Chimera 1.17 and PyMOL 2.5.5.

### 2.5. Calculation of Poisson–Boltzmann (PB) Electrostatic Potentials (EPs)

Sequential focusing multigrid calculations were used to numerically solve the nonlinear PB equation using the APBS (Adaptive Poisson-Boltzmann Solver) program [67] by means of the APBS Electrostatics plugin implemented in PyMOL 2.5.5. Three-dimensional grids with a step size of 0.5 Å for protein complexes were used which involved 257 × 193 × 257 (12,747,457) points. Common settings were T = 300 K, an 0.150 M ionic salt (NaCl) concentration, and dielectric constants of 4 for proteins, 1 for ligands, and 78.54 for water. The numerical output of the APBS was saved in OpenDX format, and PB-EPs were mapped onto molecular surfaces computed using PyMOL 2.5.5. EPs are given in units of *kT*/*e* (*k*: Boltzmann’s constant, *T*: absolute temperature, and *e*: electric unit charge).

## 3. Results

### 3.1. Structures of the REPI-Cofactor-Ligand Complexes

**Figure 2:**
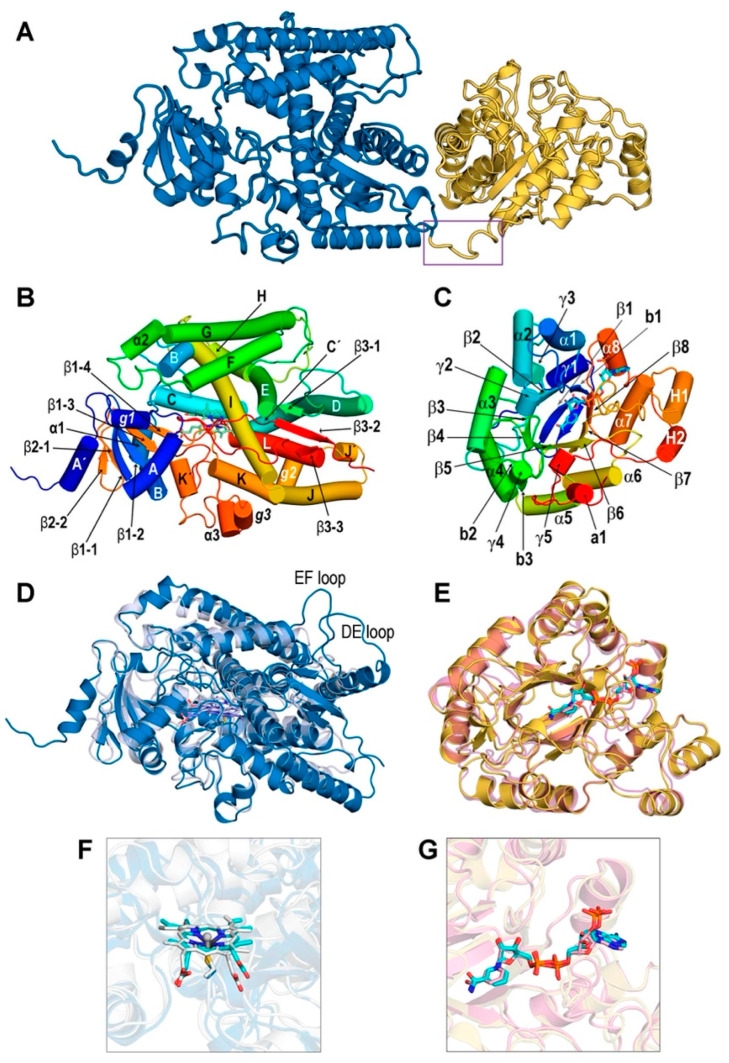
Structure of REPI. (**A**) AlphaFold model without cofactors. DRS 46–570 module in blue and DRR 571–901 module in yellow. Violet box indicates the 568–577 segment linking both modules. (**B**) Labeling of secondary structure elements in DRS according to notation in P450 enzymes [69] except α helices a1, a2, and a3, and 3_10_ helices *g*1, *g*2, and *g*3, which are either absent in other P450 enzymes or are labeled “*a*”, “*b*”, “*c*”… in the case of 3_10_ helices [69]. (**C**) Labeling of secondary structure elements in DRR was performed according to notation in COR [19] except for α helix a1 not labeled in COR and β strands not labeled (b1) or absent (b2 and b3) in COR. γ1–γ5 symbols denote 3_10_ helices not assigned in COR [19]. Rainbow coloring in both (**B**,**C**) starts at N-terminus in deep blue and ends at C-terminus in deep red. (**D**) Superposition of DRS (sky blue) including heme (sticks with violet carbons) in our optimized geometry and chain A of oxidoreductase CYP76AH3 from *S. miltiorrhiza* (light blue, PDB id. 7X2Q, [45]), including heme in the crystal geometry (sticks with light blue carbons). (**E**) Superposition of DRR (yellow) including NADP(+) in our optimized geometry (sticks with cyan carbons) and chain B of AKR4C17 from *E. colona* (light pink, PDB id. 7F7K) [48] including NADP(+) in the crystal geometry (sticks with pink carbons). DRR in (**C**,**E**) is not shown at the same scale as DRS in (**B**,**D**): their relative sizes are seen in (**A**). (**F**) Heme cofactors in a view after a 90° vertical rotation of (**D**) showing the C513.Sγ (C: deep blue)-Heme.Fe (cyan carbons) bond in DRS and the C437.Sγ (C: white)-Heme.Fe (white carbons) bond in 7X2Q. (**G**) NADP(+) cofactors in superposition (**E**).

The AlphaFold model of REPI used as initial scaffold for constructing the complexes addressed in our study shows two well-differentiated domains, DRS (residues 46–570) and DRR (residues 571–901), connected by a single unstructured segment (residues 568–577). Although the AlphaFold model places them in close proximity in space (Figure 2A and Figure S1A), the dynamics of REPI complexes reveal significant movement between both modules (Section 3.2). We discarded in our study the N-terminal region 1–45 that includes the transmembrane a-helix 14–39 along with two short, disordered segments (Figure S1A). DRS and DRR domains are clearly identified in the AlphaFold predicted aligned error (PAE) map [21] (Figure S1B). PAE maps are highly reliable identifiers of domains within proteins but not of their relative position in space [21]. The predicted local difference distance test (pLDDT) metrics [21] in the AlphaFold model structure (Figure S1C) show that the great majority of the 856 residues (46–901 sequence) are modeled with confidence that is “very high” (526 residues) or “high” (210 residues; terms qualifying confidence are those used by AlphaFold [21] and its Database [22]). This is consistent with the fact that most of the REPI sequence folds into secondary structure elements (Figure 2B,C, Table S1). The segments connecting secondary structures are modeled with “low” (56 residues) or “very low” (64 residues) confidence because of their unstructured nature, as their large motions in MD simulations discussed below demonstrate. It must be noted that the discarded N-terminal 45 residues are modeled with low (19) or very low (26 residues) confidence (Figure S1C).

To test the possible influence of updates made to learning datasets after the July 2022 release of the AlphaFold Database (AFDB) from which we downloaded the REPI initial model, we also predicted the REPI structure using AlphaFold2 (AF2) software. To this end, we used ColabFold v1.5.3 [68], which incorporates new datasets, in particular PDB100 (updated June 2023), instead of the previous PDB70 dataset (https://github.com/sokrypton/ColabFold (accessed on 4 December 2023)). The results for the five models generated using AF2 are shown in Figure S2 compared with the AFDB model. The two domains of REPI are apparent even in the sequence coverage profile (Figure S2A). The five AF2 models display nearly coincident per-residue pLDDT profiles (Figure S2B) and PAE maps (Figure S2C) showing the separate domains. The per-residue pLDDT profiles for the five AF2 models and the AFDB model are extremely similar (Figure S2D), and the global metrics of confidence provided by the mean pLDDT for the AFDB model (85.7) and the best AF2 model 3 (85.6) are nearly identical. The superposition of the separate DRS (Figure S2E) and DRR (Figure S2F) modules for the six models also reveals a close similarity except obviously in some longer mobile loops (especially DE loops) in the DRS module. These results support the reliability of the AFDB model as a basis for constructing the REPI complexes submitted to our dynamic study and demonstrate that for well-known architectures such as the P450 cytochrome (DRS) and AKR (DRR) with plenty of experimental structures in the PDB, the predictions of AlphaFold have high confidence and, as of December 2023, do not seem sensitive to recent updates to the learning datasets.

The high degrees of similarity of the DRS and DRR modules to well-studied P450 cytochromes and AKRs, respectively, allows for their secondary structure elements assigned with DSSP [19,39,69] to be labeled according to standard notation in the literature, with some exceptions (Figure 2B,C, Table S1). In addition to the helices and strands shown in Figure 2C, the COR article also proposed three loops named A, B, and C which are presumably involved in substrate specificity and cofactor binding [19]. These loops were assigned in DRR (Table S1) according to the COR-DRR structural alignment. Although not included in the labeling of Figure 2C, these loops will be considered when discussing the catalytic site of DRR (Section 3.3).

A Dali search in a non-redundant subset of the PDB with <90% sequence identity to each other (PDB90, [70]) gave similar structures for DRS 232 above the significance threshold (Dali Z-score > 2.0). Among these, eight had a Z-score > 35.0, with the top structure (Z-score = 40.5) being sugiol synthase CYP76AH3, a key enzyme in the tanshinone synthesis pathway from *Salvia miltiorrhiza* [71] in a crystal structure (PDB id. 7X2Q [45]) that includes heme. The superposition of 7X2Q with REPI-DRS (Figure 2D) shows a highly similar architecture with TM-score = 0.828 and 462 out of the 467 residues included with RMSD = 2.30 Å. The only differences are seen in the DE and EF loops, which are much shorter in 7X2Q than in DRS (Figure 2D). As for DRR, Dali found in the PDB90 set 1027 structures above the threshold, with nine entries with a Z-score > 40.0 from which the top structure (Z-score = 45.1) was COR in the crystal structure of its apo form (PDB id. 7MBF, [19]). For the sake of a full comparison of our model structure for the DRR-NADP(+) complex including a cofactor, we selected the second entry in this search: AKR4C17 from *Echinochloa colona* in a crystal structure (PDB id. 7F7K [48]) with an NADP(+) cofactor. This AKR is one of the enzymes involved in resistance to the glyphosate of *E. colona* grass and represents the first naturally evolved molecular machinery for degrading glyphosate reported in plants [48]. The similarity of this structure to REPI-DRR has Z-score = 43.3, and their superposition (Figure 2E) shows nearly identical architectures: TM-score = 0.905 and 310 out of the 331 residues in DRR included with RMSD = 1.43 Å. For the DRR-COR comparison, we found TM-score = 0.913 and the same 310 residues of DRR included in the superposition with RMSD = 1.12 Å.

It should be emphasized that the structures of the REPI complexes used for the Dali searches, TM-align comparisons, and structural alignments were those resulting from NAMD minimizations of the complexes upon the insertion of cofactors and ligands explained in the Materials and Methods section. Comparing cofactor geometries in these optimized structures and in experimental crystal structures is thus a more stringent test of reliability for these models to serve as initial structures for our MD study. This comparison is shown in Figure 2F,G for heme in DRS and for NADP(+) in DRR, respectively. Given that the structural alignments underlying this comparison refer exclusively to complete protein chains, the agreement in cofactor locations can be regarded as satisfactory. In the case of DRS, the locations of the cysteines 513 in DRS and 437 in 7X2Q that coordinate heme iron atoms (mentioned in Section 2.1) were nearly coincident.

### 3.2. General Features of MD Simulations and Final Structures of REPI Complexes

For ease of notation, the four systems introduced in Section 2.2 are herein labeled as follows: (a): “No ligand” (DRS-heme-DRR-NADP(+) complex), (b): “REN@heme” (DRS-heme-REN-DRR-NADP(+) complex), (c): “DER@heme” (DRS-heme-DER-DRR-NADP(+) complex), and (d): “DER@NADPH” (DRS-heme-DRR-NADPH-DER complex).

**Figure 3:**
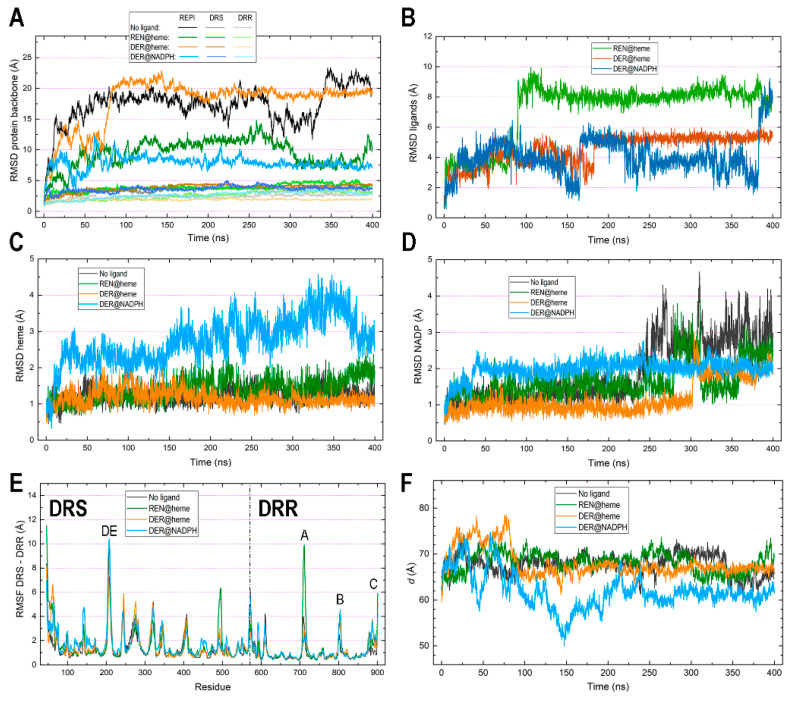
General features of 400 ns MD simulations of the four REPI complexes studied. (**A**). RMSD of protein backbone atoms. “REPI” plots correspond to the whole 46–901 protein upon aligning the complete structure, “DRS” plots correspond to the 46–570 module upon aligning the DRS structure only, and “DRR” plots correspond to the 571–901 module upon aligning the DRR structure only. (**B**) RMSD of ligand non-hydrogen atoms. “REN@heme” and “DER@heme” plots upon aligning the DRS structure only, and “DER@NADPH” plot upon aligning the DRS structure only. (**C**) RMSD of heme non-hydrogen atoms upon aligning the DRS protein structure only. (**D**) RMSD of NADP(+) or NADPH non-hydrogen atoms upon aligning the DRR protein structure only. (**E**) RMSF of the 46–570 segment (DRS) followed by RMSF of the 571–901 segment (DRR) upon aligning the DRS and DRR structures separately. The vertical dash–dot line at residue 571 divides both domains. Capital letters label some prominent peaks associated with relevant loops in DRS and DRR. (**F**) Variation in the distance between the centers of mass of the cofactors heme and NADP(+)/NADPH.

**Figure 4:**
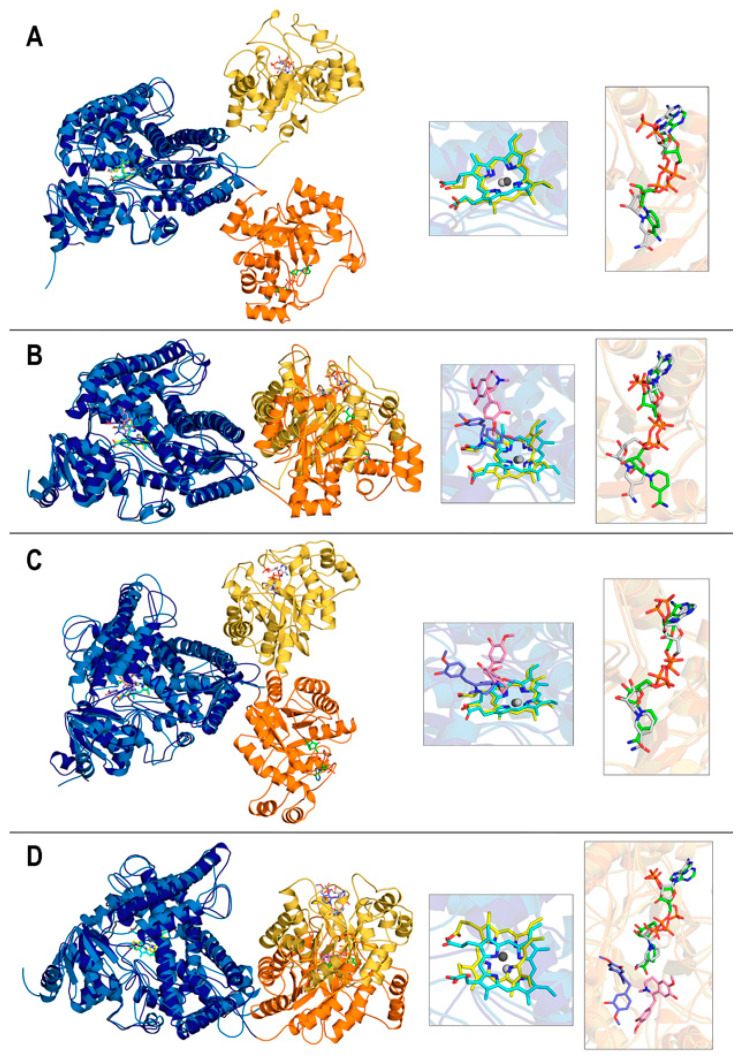
Initial and final structures of REPI complexes in the 400 ns MD simulations superimposed upon structurally aligning only the DRS domain. (**A**) “No ligand”, (**B**) “REN@heme”, (**C**) “DER@heme”, and (**D**) “DER@NADPH”. Insets on the right of protein cartoons show initial and final geometries of heme (**left** inset) and NADP(+)/NADPH (**right** inset) with cofactors and ligands at locations resulting from the corresponding structural alignments of DRS (heme) or DRR (NADP) domains. Color codes used in the four panels are as follows: Initial structures of DRS and DRR in sky blue and yellow, respectively. Final structures of DRS and DRR in deep blue and orange, respectively. Cartoons are shown with the “Smooth Loops” graphical option in PyMOL. Initial and final geometries of heme: sticks with carbons in cyan and yellow, respectively, with initial and final iron atoms shown as light gray and dark gray spheres, respectively. Initial and final geometries of NADPs: sticks with carbons in white and green, respectively. Initial and final geometries of ligand (same colors for REN and DER): sticks with carbons in slate blue and pink, respectively.

Root mean square deviation (RMSD) plots are shown in Figure 3A,B. Recall that the RMSD is the standard measure of distance between the atom coordinates of complete molecules or parts of them. In MD, the RMSD measures the average distance between a set of atoms along a simulation, typically focusing on the set of backbone atoms in the case of proteins. The RMSD thus measures how much the main chain conformation changes along a simulation. For non-protein molecules, that set usually comprises all their non-hydrogen atoms. RMSDs for protein backbones show huge differences when computed for the complete 46–901 REPI chain (Figure 3A). Considering its structure, composed of two well-defined domains linked by an unstructured short segment (Figure 2A), one should expect large conformational changes associated with the motion of one domain with respect to the other domain. However, while the two systems in which the ligand is bound to its expected site (“REN@heme” and “DER@NADPH”) show moderate–small variations, the “No ligand” and “DER@heme” systems display very large values systematically greater than 15 Å (Figure 3A). The dynamics of these two systems lead them to adopt a relative position between the DRS and DRR domains which is completely different to that of their initial structures (Figure 4). This positional change is associated with conformational freedom of the linker segment, freedom which is absent (or drastically reduced) in “REN@heme” and “DER@NADPH” and which is manifested at the very beginning of the simulations, as the rapid increases in the “No ligand” and “DER@heme” curves in the first 25 ns demonstrate (Figure 3A). In sharp contrast, when the RMSD is computed separately for the DRS and DRR domains to measure their intrinsic motion (i.e., how much they separately change with respect to their initial geometries), the plots are nearly flat, with values systematically below 5 Å (Figure 3A). This result indicates that the DRS and DRR domains are stable, almost rigid structures except for the motion associated with some of their loops (Section 3.4) which, nevertheless, does not have a large effect on the mobility of the domains. This is even more marked in DRR than in DRS (Figure 3A) because DRR (331 residues) is smaller than DRS (525 residues) and thus has fewer loops that are shorter than those of DRS (compare Figure 2D,E).

As for the RMSDs of ligands (Figure 3B), it must be first noted that both REN and DER are molecules with little conformational freedom due to the predominance of cycles in their structure. Except for -OH and -OCH_3_ groups bonded to the two aromatic rings, there is a single moiety with two adjacent fully (not only partially) rotatable bonds, one of which is just the bond connected to the asymmetric carbon involved in epimerization (Figure 1). That being said, the ligand plots show different behaviors depending on the binding site. After a similar moderate increase during the first 90 ns, both DER curves oscillate similarly between 100 and 175 ns, and then the “DER@heme” curve stabilizes at ~5.5 Å while the “DER@NADPH” curve continues to oscillate around ~4 Å, finally increasing considerably at 380 ns. On the contrary, the “REN@heme” curve suffers a marked increase at ~100 ns and then shows a flat pattern at large values ~8 Å. Interestingly, the mobility of ligands is not connected to that of cofactors. In fact, heme shows a marked change only in the “DER@NADPH” complex, while it remains quite stable in the other complexes with small RMSD values < 1.5 Å. This result indicates a strong binding in DRS which is, however, remarkably different in “DER@NADPH” (Figure 3C). NADP(+)/NADPH shows a somewhat complementary behavior to heme since the only flat curve during most of the simulation is just the “DER@NADPH” curve, while the other systems display marked changes after 250 ns (“No ligand”), 275 ns (“REN@heme”), and 300 ns (“DER@heme”). (Figure 3D). These features are addressed again below when the interaction energies are discussed in Section 3.5.

The root mean square fluctuation (RMSF) measures individual residue flexibility, that is, how much a particular residue fluctuates during the simulation. It is usually computed on the α-carbon of each residue as the RMS of the difference between its instant position and its position averaged over the whole simulation. The RMSF plots in Figure 3E were obtained separately for DRS and DRR upon aligning residues 46–570 and 571–901, respectively, in separate calculations. Except for the loops analyzed in Section 3.4, both domains exhibit very stable motions, with most residues demonstrating small fluctuations <2 Å. This result agrees with the previously mentioned fact that DRS and DRR architectures have many α or 3_10_ helices and β-strands (Figure 2B,C). This stability of both domains taken separately is maintained for the entire MD simulations, as also manifested through the maintenance of their assigned secondary structures (Figures S3 and S4). From the very beginning until the very end of the simulations, these changes in the secondary structure are small for virtually the entire sequence in both DRS (Figure S3) and DRR (Figure S4) in the four systems. Only a few short segments change from coil to helical or vice versa in some of the complexes (Section 3.4).

The separation between cofactors, measured as the distance between their centers of mass, is largely constant along the simulations in three of the four systems, with “DER@NADPH” being the exception (Figure 3F). The following average and standard deviation (in parentheses) values (Å) computed over the 400 ns simulations are 67.761 (2.142) in “No ligand”, 68.221 (2.147) in “REN@heme”, 67.972 (3.086) in “DER@heme”, and 61.975 (3.898) in “DER@NADPH”. To assess these distances, it is worth mentioning that an experimental structural and kinetic study of the fusion protein 4-coumaroyl-CoA ligase stilbene synthase found that the colocalization of the two active sites within 70 Å of each other provides the structural basis for bifunctional catalysis [72]. While the “DER@NADPH” system has a shorter distance about 62 Å (although with large deviation of ~4 Å), the other three systems show values around 68 Å, with deviations between 2 and 3 Å. This feature is directly connected with the variations revealed by the RMSD plots (Figure 3C,D), in particular with the large changes in heme in the “DER@NADPH” system which are, in turn, related to the weaker attraction energy between DRS and heme in this system *(*Section 3.5).

The final structures obtained after the 400 ns simulations are displayed in Figure 4 in the superpositions resulting from structurally aligning the DRS domains only. As noted above, the flexible segment that links the DRS and DRR modules can adopt different conformations that result in the distinct orientation of one domain with respect to the other domain in two cases (“No ligand” and “DER@heme”), while the differences are small enough to keep this orientation largely unchanged in the other two cases (“REN@heme” and “DER@NAPDH”). Note that this result is just what is described by RMSD curves for whole proteins (Figure 3A. However, the motion of one module with respect to the other module does not affect cofactors as protein domains “drag” the cofactor inside (Figure 3C,D). Also, the relatively constant distance between cofactors (Figure 3F) is neither affected because the short linker segment permits changes in the orientation of DRS and DRR but without large variations in the distances between modules, as the four panels in Figure 4 illustrate.

Additionally, the linker segment 568–577 has the sequence IKPCVQSAASE; thus, P570 in the third position introduces an additional restriction to the possible conformations that the first half of the segment can adopt. Although the differences between the initial and final structures of DRS and DRR are small, they can be quantified. Their structural alignments obtained using TM-align gave the following TM-scores for systems (a)–(d), respectively, 0.895, 0.881, 0.862, and 0.871 for DRS, and 0.916, 0.911, 0.904, and 0.896 for DRR. If one recalls that TM-scores closer to 1 indicate greater structural similarity, it is apparent that DRR modules change less than DRS modules and that the absence of a ligand in system (a) implies fewer variations.

As for the comparison between the initial and final geometries of cofactors and ligands, the insets in Figure 4 reveal that heme and NADP(+)/NADPH geometries keep their relative positions and only vary their relative orientations, especially in the systems with larger cofactor RMSD values (Figure 3C,D). Thus, “DER@NADPH” has heme with slightly greater variations (Figure 4D) and “REN@heme” has NADP(+) with a noticeable change in the position of its nicotinamide ring (Figure 4B). In contrast, ligands exhibit in the three systems much more marked changes in both position and orientation.

### 3.3. Catalytic Sites of REPI

#### 3.3.1. REPI-DRS

**Figure 5:**
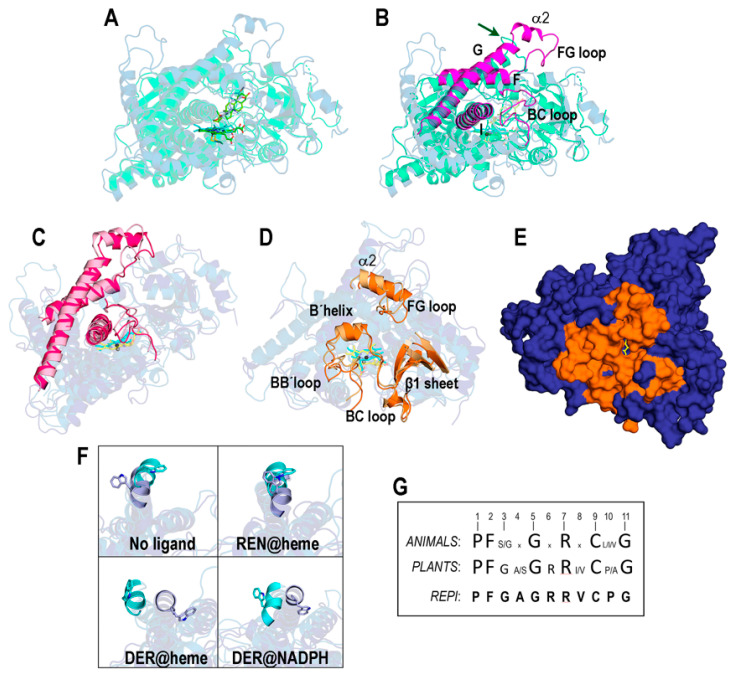
Structural features of REPI-DRS. (**A**) Superposition of the model initial structure of “REN@heme” (blue) and the crystal structure (PDB 6A15) of *Arabidopsis* CYP90B1 (green) in complex with cholesterol [73]. Heme cofactors are sticks with carbons in cyan (“REN@heme”) and green (6A15). Ligands are sticks with carbons in light blue (REN) and lime green (cholesterol). (**B**) Superposition in (**A**) highlighting in the “REN@heme” model the conserved secondary structure elements involved in housing heme (magenta). The arrow indicates the FG loop in the crystal structure lacking the α2 helix that is present in REPI-DRS. (**C**) Superposition of initial and final structures in the 400 ns simulations of “REN@heme”, highlighting the secondary structure elements marked in (**B**). Initial structure: sky blue, sticks with cyan carbons for heme, highlighted elements in light pink. Final structure: deep blue, sticks with yellow carbons for heme, highlighted elements in deep pink. (**D**) Same as (**C**), highlighting the secondary structure elements of pathway 2a (light orange and deep orange for initial and final structures, respectively). (**E**) Surface of the final structure in (**D**) showing the pocket cavity (same color codes as in (**D**)). (**F**) Initial and final geometries of W278 and the α2 helix (cyan and light blue, respectively). (**G**) Consensus sequence of the heme motif in animals [74,81], plants [82], and REPI cytochrome P450 enzymes. Letter size denotes the degree of sequence conservation, with the largest size indicating totally conserved residues and “x” meaning “any residue”.

We first show in Figure 5A,B a comparison between our initial optimized model structure of the DRS-heme-REN complex and an experimental structure for a plant P450 cytochrome complex: the crystal structure of an *Arabidopsis* CYP90B1-cholesterol complex (PDB id 6A15, [73]). Although both structures are highly similar (TM-score = 0.860), some significant differences are noted in loops that do not affect the good agreement between the model and crystal geometries of both heme and ligands (Figure 5A). These differences are particularly apparent in the FG loop, which plays an essential role in controlling access to the active site of P450s. In CYP90B1, this loop has 10 residues (217–226) with sequence VSAPLNLPGT (indicated by the arrow in Figure 5B) so that the two nearby prolines restrict the conformational flexibility of the whole loop. In contrast, the FG loop in REPI-DRS has the 19 residues (266–284), TSPVSDNVPMLG*WIDQL*TG, in which the five ones shown underlined and in italics adopt a helical conformation (α2 in Figure 2B and Figure 5B and Table S1) with coil segments at both ends (Figure 5B). Even though the FG loop in REPI-DRS also has two prolines, they are much further from each other than in CYP90B1. These structural features confer the FG loop in REPI-DRS considerable flexibility. It must be highlighted that the α2 helix is not present in customary assignments of a secondary structure in cytochrome P450s [39,69].

The comparison between the initial and final structures of the “REN@heme” system (Figure 5C,D) shows that the MD simulation introduces no significant changes in the elements of the secondary structure involved in controlling access to the binding site. These elements, highly structurally conserved in crystal structures of P450s [39,74], are the I helix with the segment that harbors heme and the F and G helices and the BC and FG loops (Figure 5B,C). This structural conservation was not observed in all the REPI systems studied herein, as the analysis of the FG loop reveals (Figure S5A). On one hand, the secondary structure composition of the FG loop is maintained in all systems except “DER@NADPH”. On the other hand, its spatial position is held in “REN@heme”, slightly less in “No ligand”, and far less in “DER@heme” (Figure S5A). It seems, therefore, that the dynamics of the DRS domain are sensitive to the ligand (or lack thereof) at the catalytic site. However, there is a large diversity of other elements involved in substrate access to the heme site that are highly variable in sequence and structure in cytochromes P450 [39,74,75]. The channel access most frequently found in MD simulations of P450s is known as pathway 2a [74,75], considered “the most likely route for substrate access and product egress” [39]. Pathway 2a consists of a B’helix, β1 sheet, and FG, BB’, and BC loops, also maintained with very little variation in the simulation of the “REN@heme” system (Figure 5D). In the final structure of this complex, the channel that connects the aqueous outer environment with the heme site in this complex can be seen (Figure 5E).

An interesting feature of REPI-DRS regarding the α2 helix mentioned above is the position of the exposed W278 residue at its N-terminal end. The uniqueness of tryptophan and the relevance of its special properties in biology have been the subject of intense research [76,77]. In particular, exposed tryptophans do play key roles in the dynamics of amino acids at protein surfaces [78] and in mechanisms of opening and closing grooves or cavities that harbor ligands of disparate chemical natures (for representative examples, see Refs. [79,80]. The panel in Figure 5F displays the relative location of W278 in the α2 helix in the initial and final structures of the four systems under study. According to the above analysis of the relative position of the FG helix (Figure S5A), the position of W278 suffers almost no change in only the “REN@heme” complex, whereas it shows noticeable variations in the remaining complexes (Figure 5F).

A comparison of the 11-residue sequence of the heme motif in REPI-DRS (505–515) with the consensus sequence of this motif in animals [74,81] and plants [82] reveals a high degree of conservation with animals and perfect conservation with plants if one of the two most frequent amino acids in positions 4, 8, and 10 in the plant consensus sequence is considered (Figure 5G).

As for the position of substrates at the heme catalytic site, the crystal structures of several cytochrome P450 complexes in plants exhibit a wide diversity of spatial locations. Our model structures for the “REN@heme” and “DER@heme” systems show that the REN and DER ligands are within the experimental range of representative crystal geometries [73,83] (Figure S5B).

#### 3.3.2. REPI-DRR

**Figure 6:**
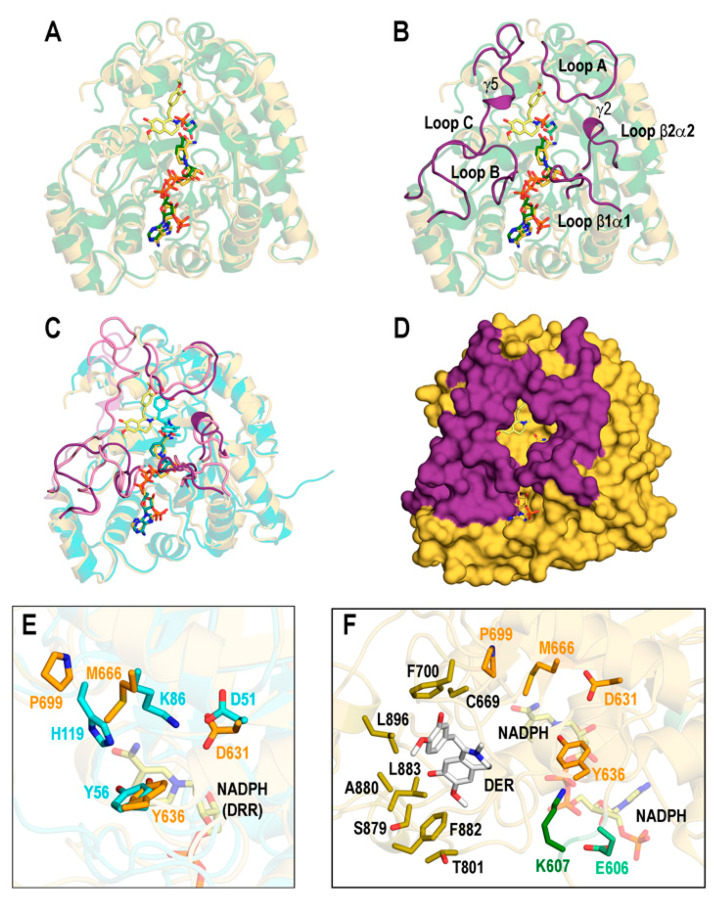
Structural features of REPI-DRR. (**A**) Superposition of model final structure of “DER@NADPH” (light orange) and the crystal structure (PDB id 7F7M) of AKR4C17 (green) in complex with glyphosate [48]. NADPH in “DER@NADPH” and NADP(+) in 7F7M are the sticks with carbons in light orange and green, respectively. Ligands DER and glyphosate in 7F7M are the sticks with carbons in yellow and light green, respectively (**B**) Superposition in (**A**) highlighting in “DER@NADPH” the secondary structure elements (violet) involved in forming the substrate-binding pocket (loops A and C), cofactor and substrate binding (loop C), and contributing to substrate specificity and catalysis (loops β1α1 and β2α2), according to [19]. (**C**) Superposition of initial and final structures in the 400 ns simulations of “DER@NADPH” highlighting the secondary structure elements marked in (**B**). Initial structure: light blue, sticks with greenish blue carbons for NADPH and cyan carbons for DER, highlighted elements in light pink. Final structure: same graphical options as in (**B**) except loop C, which is purposely left semitransparent to highlight the disappearance of 3_10_ helix γ5. (**D**) Surface of the final structure in (**C**) showing the pocket cavity (same color codes as in (**B**)). (**E**) Active site in the structural superposition of COR and the final structure of “DER@NADPH”. The cofactor shown as sticks with yellow carbons is NADPH in “DER@NADPH” (the crystal structure of COR, PDB id 7MBF [19], has neither cofactor nor ligand). The catalytic tetrad observed in AKRs [19] is shown with cyan carbons in COR, and the structurally equivalent residues in “DER@NADPH” are shown with orange carbons. (**F**) Binding site in the final structure of “DER@NADPH” (light orange). DER substrate is shown with white carbons, and residues in a neighborhood of 4 Å are shown with carbons in an olive color. The residues equivalent to the catalytic tetrad shown in (**E**) are included (orange carbons), as well as the E606 (light green carbons) and K607 (deep green carbons) residues discussed in the text.

AKR-like structures similar to DRR rarely include both a NADP cofactor and substrate. In fact, among the nine PDB entries with a Dali Z-score > 40.0 in the comparison with REPI-DRR, only one structure, AKR4C17 from *E. colona* (PDb id 7F7M) [48], had both NADP(+) and a substrate. The structural comparison of “DER@NADPH” with 7F7M in Figure 6A shows good agreement in the geometries of the NADP(+) and NADPH cofactors, and substrates (note the similar spatial positions of the N atoms in both DER and glyphosate). However, we considered the structural study of COR [19] as the basis for our comparative analysis because that study provides valuable insights into AKR function in processing compounds similar to reticuline. In this regard, we took the secondary structure elements for substrate specificity indicated in [19], keeping the same notation (which is used in Figure 6B). Those elements flank the central TIM-barrel (α1–α8 helices plus β1–β8 strands, Figure 2C, Table S1) of AKRs and are as follows: loops A and C that contribute to forming the substrate-binding pocket, loop B that contributes to both cofactor and substrate binding, and loops β1α1 and β2α2 that also contribute to specificity and binding and include two of the four catalytic residues (631 and 636) in DRR addressed below. While these elements are assigned as coils in COR, DRR displays 3_10_ helices γ2 and γ5 inserted in loops β2α2 and C, respectively (Figure 6B). The dynamics change the spatial locations of all these elements in the “DER@NADPH” structure little except the 3_10_ helix γ5 in loop C, which disappears (Figure 6C). The protein surface shows quite clearly the appropriateness of the elements proposed in the COR study as likely involved in the formation of the binding pocket in REPI-DRR (Figure 6D).

The catalytic tetrad “seen in all AKRs” [19] formed by D51, Y56, K86, and H119 in COR is not conserved in DRR (Figure 6E). While the aspartate–tyrosine pair, close in sequence and in structure, is present in DRR (D631 and Y636) at nearly coincident spatial locations with respect to COR, the lysine–histidine pair is not. Instead of these two COR amino acids, DRR has M666 and P699 at the sequence and structure locations of COR K86 and H119, respectively (Figure 6E). Based on a sequence analysis and on a homology model of the DRR structure, the COR study discussed this difference and pointed out that “the proton transfer steps in the canonical AKR mechanism cannot occur in DRR” [19] because M666 and P699 lack titratable protons. It should be clarified that the DRR sequence numbering used in the present work is one unit greater than the DRR sequence numbering used in the COR report. The reason for this discrepancy is that the AlphaFold model underlying our structural analyses covers the complete REPI sequence in which M1 of the DRR module is actually M581 of the complete REPI protein. Thus, D51 in COR is D631 in DRR, et cetera. However, the authors of the COR study mentioned that “Glu-605” in DRR (their numbering) “that is predicted to be close to the highly conserved Tyr-635 residue (albeit on the opposite face)” [19] could adopt a role alleviating steric hindrances in the active site like that of an additional catalytic glutamate observed in some AKRs. That residue is E606 in Figure 6F in which we display the binding site defined by a neighborhood of 4 Å around DER in the final structure of the “DER@NADPH” system after the MD simulation. It is apparent that, in fact, E606 is close to Y636 in our final structure, but note that there is a contiguous lysine (K607 in Figure 6F) not mentioned in the COR study that might also be relevant in this analysis.

Furthermore, we suggest that K607 could participate in the catalytic activity of DRR to a (likely) greater extent than E606 for two reasons. First, because K607 could be a substitute for COR-K86. Second, because as far as the final structure of “DER@NADPH” is concerned, K607 is closer to NADPH than E606; the distance from N atom of the nicotinamide ring in NADPH to the N atom of K607 is 5.9 Å, whereas that distance to the closer O atom of E606 is 10.8 Å. This suggestion might be supported by the DER binding site shown in Figure 6F, a site different to that considered by the authors of the COR report as representative of typical AKRs (Figure 8 in [19]), although both F700 and F882, seen in Figure 6F, are also present in their binding sites (H120 and F302 in the COR protein).

### 3.4. Interaction between DRS and DRR Domains

**Figure 7:**
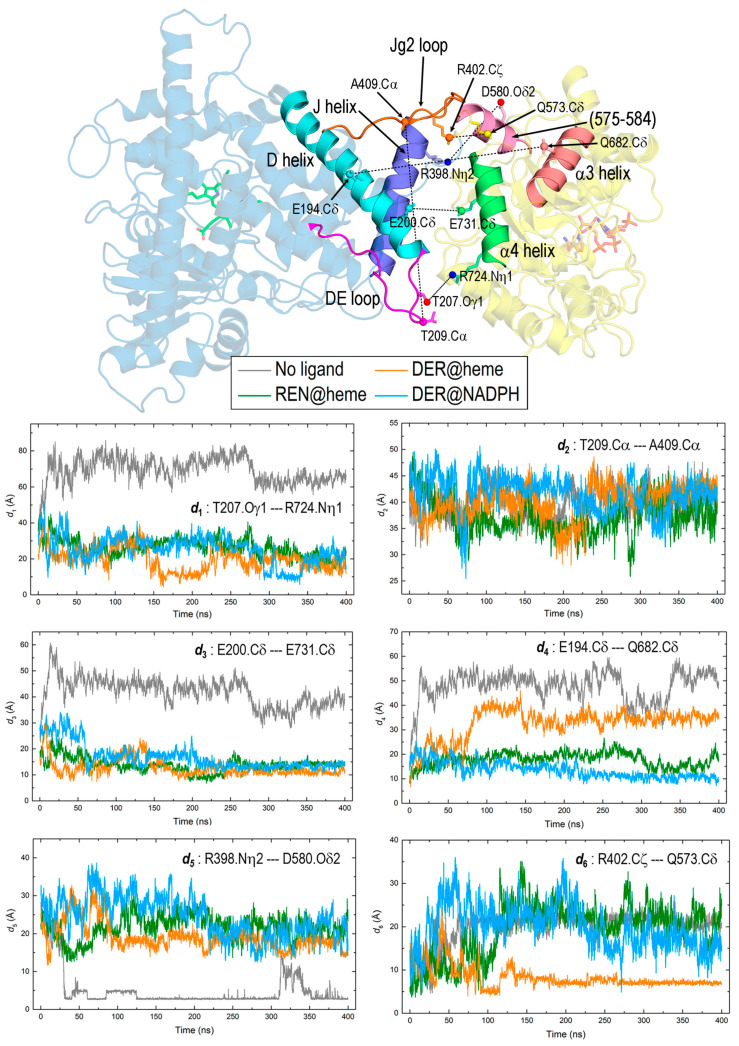
Interdomain distances selected to monitor DRS-DRR interactions along the 400 ns MD simulations. Top: final structure of the “DER@heme” complex used as a scaffold to define the distances. Blue: DRS; yellow: DRR; heme and NADP(+): sticks with carbons in green and white, respectively. The selected structural elements used to monitor DRS-DRR interactions, shown in different colors, are grouped in the following pairs: (1) DE loop (DRS)—α4 helix (DRR), (2) DE loop (DRS)—Jg2 loop (DRS), (3) D helix (DRS)—α4 helix (DRR), (4) D helix (DRS)—α3 helix (DRR), (5) J helix (DRS)—segment 575–584 (DRR), and (6) residues R402 (DRS)—Q573 (DRR). The corresponding distances were computed between the pairs of atoms indicated in the top cartoon, and the labels of the six plots in the panel that show their variation along the simulations.

The dynamics of REPI are primarily manifested in the relative motion of the DRS and DRR domains which, in turn, leads to minor but essential internal changes regarding structural details (discussed in the preceding sections) and interaction energies (addressed in Section 3.5). After analyzing the MD trajectories of the four systems to identify which parts of the structure clearly vary to a greater extent than the remaining parts, we selected the structural elements shown in Figure 7 to monitor interdomain changes. This monitoring was carried out by analyzing along the MD simulations the following six pairs: (1) DE loop (DRS)—α4 helix (DRR), (2) DE loop (DRS)—Jg2 loop (DRS), (3) D helix (DRS)—α4 helix (DRR), (4) D helix (DRS)—α3 helix (DRR), (5) J helix (DRS) segment 575–584 (DRR), and (6) the amino acid pair R402 (DRS)—Q573 (DRR). The 575–584 segment of DRR was assigned a coil in the original AlphaFold structure used for labeling the secondary structure along the article (Figure 2B,C, Table S1), but the conformational backbone dihedral angles in those residues were so close to α-helical values that they frequently arranged in transient α-helices along the simulation. This is indeed the case for the final structure of the “DER@heme” complex used for displaying the selected structural elements in Figure 7. Following the selection of these elements, we analyzed the behavior of their residues to identify those most sensitive to interdomain changes in the dynamics of the four complexes. This analysis led us to choose the six atom pairs presented in Figure 7 to compute the variations in their distances along MD simulations (panel in Figure 7). Although pair (2) involves two atoms within the same DRS domain, we included their distance because it probes the separation between the two loops of DRS that have the greatest motions along simulations: DE and Jg2. With this distance, we intended to show that despite their large displacements, both loops maintain a similar separation in the four systems (panel *d*_2_), a result that could be interpreted as if those loops moved in a coordinate fashion. Regarding this, note that while pair (1), which involves the DE loop, shows far greater distances for the “No ligand” system than for the other systems (panel *d*_1_), pair (5), which involves Jg2 loop, shows the opposite behavior (panel *d*_5_). However, in both cases, the distances for the complexes with a ligand show a remarkably similar pattern, which suggests that the mentioned coordinate motion of the DE and Jg2 loops would be an effect of the presence of ligand.

Pairs (1), (3), and (4), which involve the DE loop or D helix of DRS and the α4 or α3 helix of DRR, exhibit clear differences depending on the absence or presence of a ligand, irrespective of its binding site. The “No ligand” curves in panels *d*_1_, *d*_3_, and *d*_4_ have systematically longer distances than the remaining curves. However, the closer proximity between DRS and DRR structural elements in the complexes with REN or DER is particularly significant in pair (3). This pair probes the separation between the α-helices D in DRS and α4 in DRR which, as far as the distance selected measures the separation between the C-terminal end of the long D helix and the center of α4 helix, is on one side, very short, and on the other side, nearly identical in the three complexes with a ligand (panel *d*_3_). This result indicates that both helices, essential in the interdomain interaction, behave rather steadily in the simulations only when REPI bears a ligand. As for pair (1), it probes the separation between the DE loop and α4 helix too; despite the large spatial displacements of the DE loop (see below), its distance to the same α4 helix of DRR exhibits a similar behavior to that of a stable and relatively rigid element such as the D helix: (compare panels *d*_1_ and *d*_3_). Moreover, although the values in the “No ligand” curves are considerably larger in panel *d*_1_ (see the Y-axis scale) than in panel *d*_3_, the values in the remaining curves are only slightly larger in panel *d*_1_. This result suggests that the presence of a ligand stabilizes the motion of the mobile elements in DRS.

Interestingly, the “DER@heme” curve in pair (4) shows a different qualitative behavior with respect to the remaining curves of this complex in other pairs that involve helices or loops of DRS. Since pair (4) probes the distance from the DRS-D helix to the DRR-α3 helix, which is more distant from DRS than the α4 helix (cartoon in Figure 7), this result suggests that when DER is placed at the heme site instead of the NADPH site, the DRR domain moves with respect to the DRS domain with a greater displacement. If one compares the “DER@heme” system with the other complexes with ligand, the latter remark is supported by (a) the much larger RMSD of the whole REPI protein in “DER@heme” (Figure 3A) and (b) the qualitatively different spatial arrangements of the two domains in its final structure after the simulation (Figure 4). Regarding these two features, the MD results reveal that the dynamic behavior of the “DER@heme” system is more like that of the “No ligand” system than that of the other two complexes with the ligand at their expected binding sites.

This resemblance in the dynamics of the “No ligand” and “DER@heme” systems is also observed in the variation in the distances associated with pairs (5) and (6). Pair (5) involves R398 and D580, two amino acids that may present an electrostatic charge–charge attraction (an “ionic pair” in chemistry or a “salt bridge” in biochemistry) at proper distances. This ionic pair is observed in the dynamics of “No ligand” being formed at about 30 ns and remains steady with some oscillations between ~310 and 350 ns (panel *d*_5_). Pair (6) involves R402 and Q573, two amino acids that may present an electrostatic charge–dipole attraction at proper distances, which happens in the “DER@heme” complex, at ~90 ns, followed by remaining steady after ~150 ns (panel *d*_6_). Incidentally, the comparison between the “No ligand” curve in panel *d*_5_ and the “DER@heme” curve in panel *d*_6_ illustrates the stronger charge–charge interaction (shorter distance) well compared with the weaker charge–dipole interaction (longer distance). In the absence of these electrostatic attractions, the amino acid pair interactions probed by pairs (5) and (6) show only the fluctuations expected for the relative motions of the DRS and DRR domains without other features worth mentioning.

To end this section, we display in Figure S6 four snapshots of one of the trajectories that illustrate the large spatial displacement of the DE loop in DRS, an issue which is addressed below in Section 3.6 and Section 3.7 regarding dynamic structural changes potentially related to the possible channeling of a DER intermediate. In the example shown in Figure S6 (“DER@heme”), the DE loop adopts an open conformation during the first ~160 ns, starts to close at ~170 ns, remains closed up to ~230 ns, opens again at 240 ns, and remains open until the end (400 ns) of the simulation.

### 3.5. Protein–Cofactor and Protein–Ligand Interaction Energies and Water Effects

**Figure 8:**
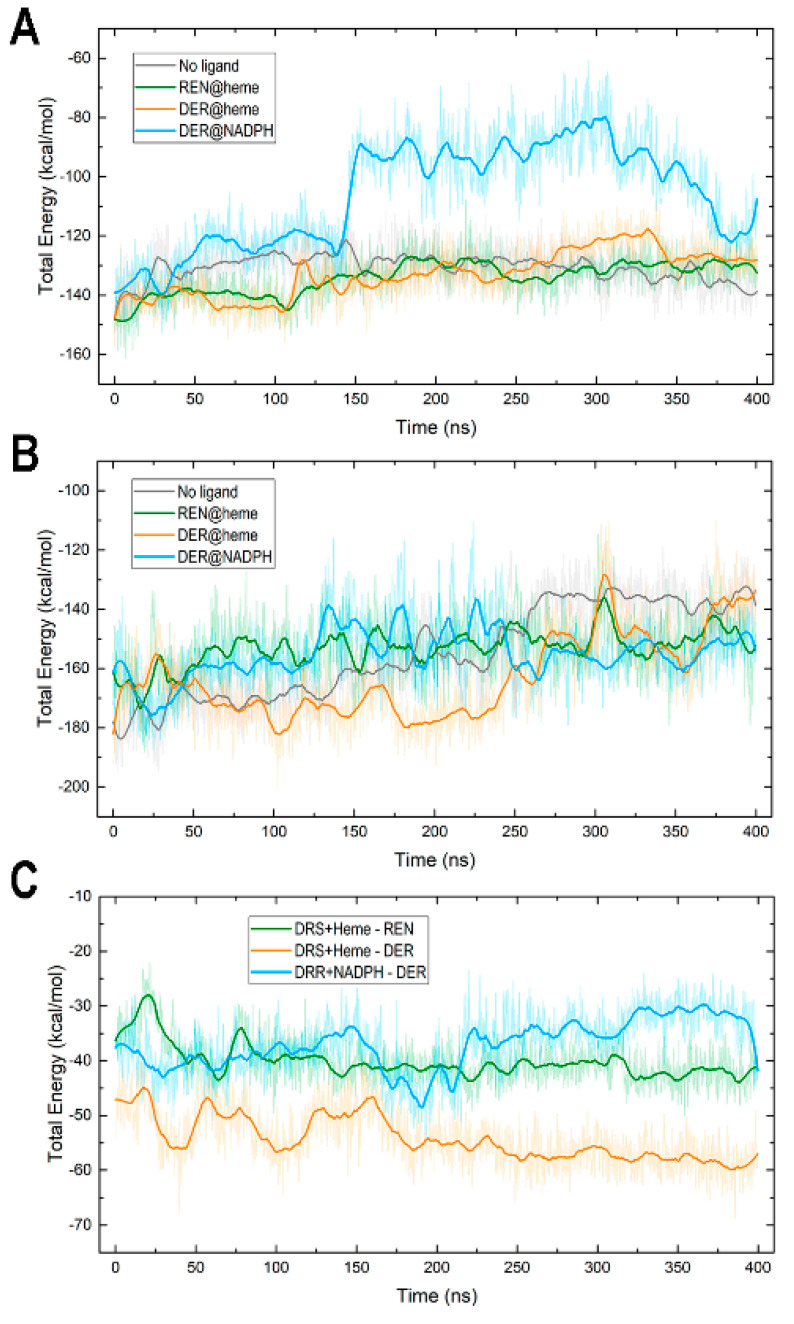
Variations along MD simulations of total non-bonded interaction energies between DRS and heme (**A**), between DRR and NADP(+) or NADPH (**B**), and between DRS + heme and REN or DER and between DRR-NAPDH and DER (**C**). Solid curves: 101-point third-order Savitzsky–Golay smoothing polynomials.

We computed variations in interaction energies along the 400 ns simulations between REPI domains and their cofactors and between REPI–cofactor complexes and their ligands. The results are plotted in Figure 8, and the average values calculated over the trajectories are given in Table 1. These interaction energies are the total non-bonded energies in the CHARMM 3.6 force field used in our work, that is, the sum of electrostatic and van der Waals energy terms. We also analyzed the effect of water around cofactors and ligands by calculating the variation in the number of (a) hydrogen bonds (HBs) between water molecules and cofactors or ligands (Figure S7) and (b) water molecules at 5 Å from cofactors (Figure S8). The values of both numbers averaged over the trajectories with their standard deviations are listed in Table 2.

The interaction between the DRS domain and its heme cofactor shows a rather similar variation pattern (Figure 8A) and very similar average values (Table 1) in all systems except “DER@NADPH”. In this complex, the DRS-heme attraction is like in the other complexes until about 144 ns, the time at which it weakens (turns less negative), increasing abruptly from −132 kcal/mol at 144 ns to −91 kcal/mol at 146 ns. However, this sudden increase is due only to the weakening of the electrostatic contribution because the VdW contribution remains steady over the whole simulation (Figure S9). This change is related to the increase in the number of water molecules that enter the active site cavity in DRS and is manifested in the greater number of water–heme HBs (Table 2, Figure S7A) and of water molecules in the vicinity of heme (Table 2, Figure S8A) in “DER@NADPH” compared to the other three systems. Despite the similarity in mobility between the DRS and DRR domains in this complex (Figure 3A), the lack of a ligand at the heme site makes the entrance to the cavity in DRS remain more open compared to when a ligand is inside (see, for instance, its small opening in “REN@heme” in Figure 5E). In “DER@NADPH”, this effect provokes, in turn, the distinct behavior noticed above in the RMSD of heme in this complex, which is far greater than that in the other complexes (Figure 3C).

When the heme site is occupied with REN or DER or when there is no ligand, the attraction energy between the DRS module and heme as well as the interactions of heme with water are strikingly similar (Table 1 and Table 2, Figure 8A, Figures S7A and S8A). Heme is located well inside a pocket in DRS (see for example Figure 5E) and consequently, the number of water molecules finding their way to heme is small, especially in “DER@heme”. Since the DER charged intermediate is located at the heme site in this complex with an attraction energy far lower than the cofactor–ligand energies in the other complexes (Table 1, Figure 8C), DER blocks water’s access to heme, the cavity that harbors heme is smaller, and the number of water molecules surrounding the heme is also smaller (Figure S8A) than in the remaining complexes.

In contrast, the interaction between the DRR module and NADP(+) or NADPH shows no great differences among complexes (Figure 8B). Energies for this interaction are larger (more negative) than those between DRS and heme (Table 1), an expected result if one considers that although the cofactors have nearly identical numbers of atoms (73 in heme and 74/75 in NADP(+)/NADPH), the chemical structures allow NADP molecules to interact with a greater number of amino acids in DRR than heme in DRS. The interaction of NADP(+)/NADPH with water also exhibits distinct features to those shown by heme because the structure of NADP is more exposed to solvent than heme (see, for example, Figure 6D), and both the number of HBs with water and the number of water molecules surrounding this cofactor are markedly greater than the corresponding values for heme (Table 2).

As for interactions with the substrates, the plots in Figure 8C and average values in Table 1 reveal similar patterns in “REN@heme” and “DER@NADPH”, whereas a much greater attraction was found for “DER@heme”. Energy similarity in the two complexes in which the substrate is located at its expected site reveals that the two chemical modifications required by reticuline epimerization seem to a similar energetic costs, a result consistent with the bifunctional nature of REPI. To rationalize the different energy in “DER@heme”, it must be highlighted that DER is the product, not the substrate, of DRS. The intention of including this complex in our MD study was to explore the dynamics of the DRS domain when its substrate REN has been already modified to DER. Given that this modification implies the appearance of a positive charge in DER, the greater attraction found in the simulation of “DER@heme” indicates that without a proper conformational change for expelling or channeling the intermediate occurring simultaneously to the transfer of electrons and protons (and O_2_ as well) associated with cytochrome activity, the cationic compound would remain bound to the heme site. Since the study of electron and proton transfer together with the REN/DER bond reorganization involves quantum calculations, this issue remains beyond the scope of the current work in which we are presenting a first structural study of the dynamics of the REPI protein. In any case, such further research should address the results unveiled by our MD study.

In conclusion, let us mention that the ligand–water interaction, irrespective of whether the ligand is REN or DER or whether the binding site is a heme or NADPH site, is scarcely significant. On average, a single HB with water (Table 2 and Figure S7C) indicates that both REN and DER are shielded from water when bound to DRS or DRR.

### 3.6. Exploration of Tunnels in REPI

**Figure 9:**
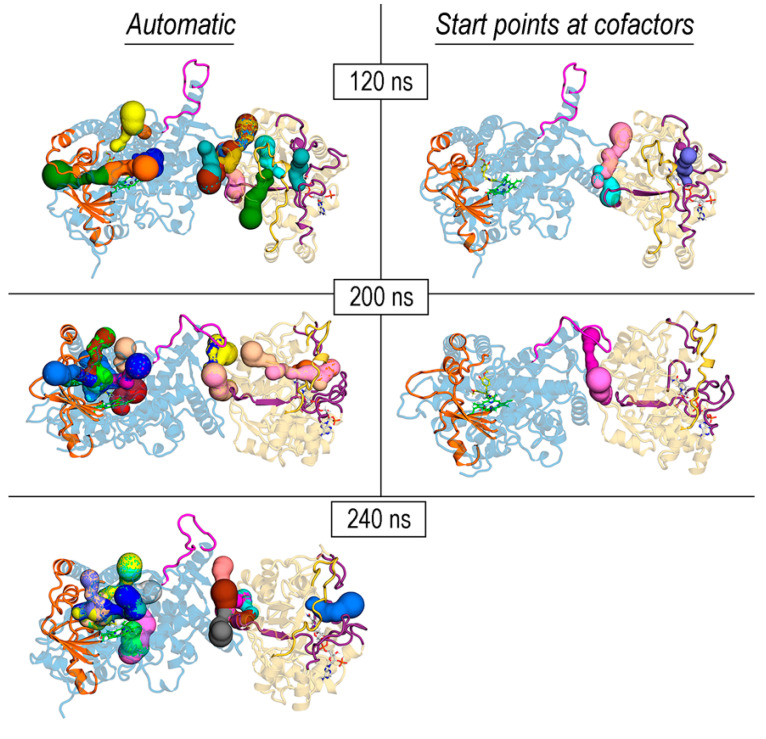
Tunnels found using MOLE 2.5, using the “automatic” selection of start points (left column) and “user-defined start points at the cofactors” heme and NADP(+) (right column) in three intermediate structures of the 400 ns simulation of “DER@heme”. Tunnels in the “Start points at cofactors” column were found only when both heme and NADP(+) cofactors were selected together as start points. This option identified no tunnels in the 240 ns structure. DRS and DRR domains are colored sky-blue and light orange, respectively. Secondary structure elements involved in cavity formation and binding selectivity in DRS (Figure 5) and DRR (Figure 6) are shown in orange and violet, respectively. Sticks with carbons in green and white represent heme and NADP(+) cofactors, respectively. The DE loop of DRS showing an open (120 and 240 ns) or closed (200 ns) conformation is colored magenta. Tunnels were colored by MOLE 2.5 according to its prescription to distinguish them in the same region of space.

The search for cavities, channels, or tunnels in cytochromes P450 has received considerable attention over the years to identify different pathways to access the active site [38,39,74,75]. In REPI, the product of the P450 module (DRS) is the intermediate 1,2-dehydroreticuline cation, which is known to protonate and tautomerize to enamine in water [15,29]. It therefore must be somehow channeled from the heme site in DRS to the NADPH site in DRR to shield it from water. For this reason, we focused on the search for tunnels/channels connecting both sites using MOLE 2.5 software [37]. This program can identify tunnels in a protein structure using a predefined set of options, or these can be tailored with user-defined selections, e.g., choosing specific start points that are fed to the MOLE algorithm [35,37]. These start points can be the 3D coordinates of space points or a selection of one or several residues or cofactors. Both automatic and user-defined options were employed in our exploration of the four REPI systems, selecting in the second option start points defined by heme in DRS and NADP (either NADP(+) or NADPH) in DRR. While the automatic detection usually returns many cavities or tunnels with unequal relevance, the user-defined-based detection performs a selective search able to find tunnels that run through specific parts of the protein. In our case, the selection of heme or NADP or both as start points allowed for the appropriate fine-tuning of the tunnels identified in the automatic mode. However, we also present the solutions found in the automatic mode because it is informative to see how the density of the tunnels changes in the different REPI systems (Figure 9 and Figure S10). For example, in automatic mode, all systems display, in both the DRS and DRR domains, a concentration of tunnels that connect the cofactor location with the exterior through the secondary structure elements involved in pocket opening and binding selectivity presented in Section 3.3 (protein segments colored orange and violet in DRS and DRR, respectively, in both Figure 9 and Figure S10). In DRS, these tunnels are related with the different pathways proposed in structural studies of P450s [39,74,75]. Tunnels in the interdomain space are only found in automatic mode in complexes with a ligand, either REN or DER (Automatic column in Figure S10).

With respect to the tunnels found in the user-defined mode (“Start points at cofactors” column in Figure 9 and Figure S10), the “No ligand”, “REN@heme”, and “DER@NADPH” systems show one single tunnel connecting heme with the solvent in DRS and another one connecting NADP(+) or NADPH with the solvent in DRR (Figure S10). In the case of the “DER@heme” system, tunnels were only found when both heme and NADP(+) cofactors were selected together as start points (Figure 9). In fact, only “REN@heme” and “DER@heme” showed a tunnel in the interdomain space, whereas “No ligand or “DER@NADPH”, each of which already has the intermediate at “its” active site in DRR (thereby not requiring it to be channeled from DRS), had no interdomain tunnel (Figure 9 and Figure S10).

To complement at the discussion at the end of Section 3.4 concerning the motion of the DRS-DE loop provoking changes between open and closed conformations in the interdomain region in “DER@heme”, we display in Figure 9 the tunnels detected in the same three intermediate structures at 120, 200, and 240 ns considered above (Figure S6). In agreement with the above comments about the strong similarity of structures at simulation times from ~240 ns until the end, tunnels found for the final structure (400 ns) are essentially the same as those found for the structure at 240 ns and are thus not shown in Figure 9. Tunnels in this system deserve a separate analysis as it is the complex in which some kind of substrate channeling was expected given that the DER intermediate is placed at the heme site where it is produced. The results displayed in Figure 9 summarize the tunnels found in the structures showing the main dynamics changes concerning the open and closed conformations of the DE loop observed in the simulation of “DER@heme”. These structures were sampled in the MD trajectory at 10 ns intervals during those opening and closing events. Tunnels detected in automatic mode are not particularly sensitive to the conformation of the DE loop, although it must be noted that the three structures in Figure 9 show more than one tunnel in the interdomain space.

In the user-defined option (“Start points at cofactors” column in Figure 9), this “DER@heme” system shows a result worthy of analyzing. Firstly, tunnels were found only when both heme and NADP(+) cofactors were selected as start points, whereas no tunnel at all was found when either heme or NADP(+) was singly selected. Secondly, a tunnel in the interdomain space was detected only when the DE loop had its closed conformation (200 ns structure in Figure 9), whereas that tunnel disappeared when the DE loop returned to its open conformation. Thirdly, no tunnels were found in either of the three possibilities for selecting start points at cofactors from 240 ns until the end (400 ns) of the simulation. This result led us to hypothesize that the formation of an internal channel in REPI that could permit 1,2-dehydroreticuline to migrate from DRS to DRR might require a closed conformation for the DE loop in DRS. However, although the interdomain tunnel in the 200 ns structure seen in the “Start points at cofactors” column in Figure 9 runs near DRR and under the DE loop of DRS, it is not a channel connecting the heme and NADPH sites. This way, as far as the structures along the simulation are concerned, the requirement of having the DE loop in a closed conformation to provoke the formation of some type of channel/tunnel connecting the DRS and DRR active sites might be necessary but not sufficient. In this regard, the formation of a tunnel for ammonia transfer in GPATase associated with conformational changes in a flexible loop between the two catalytic domains of this bifunctional enzyme [25] is a particularly relevant example. One could speculate that a conformational change in DRS provoked by the catalytic conversion of REN into DER would assist in opening an interdomain space flanked by the closed DE loop. To explore this possibility requires incorporating quantum calculations to the dynamic study of REPI for addressing structural changes associated with proton and electron transfer. This issue, which is beyond the scope of the current work, will be the subject of our forthcoming research on REPI.

### 3.7. Poisson–Boltzmann (PB) Electrostatic Potential (EP) in REPI Complexes

Most well-studied cases of substrate channeling in bifunctional enzymes occur through tunnels that connect active sites [25,84] (for a recent report, see [85] and the references therein). However, the concept of electrostatic channeling was proposed for describing the shuttling of charged intermediates across electrostatic “highways” in the surface of proteins [86,87]. In 1996, this channeling described how a negatively charged dihydrofolate intermediate moves along a positive electrostatic highway that links the two active sites in the crystal structure of a bifunctional thymidylate synthase–dihydrofolate reductase in which no tunnel was apparent. The proposal was supported by experimental kinetic analyses and Brownian dynamics simulations [87].

**Figure 10:**
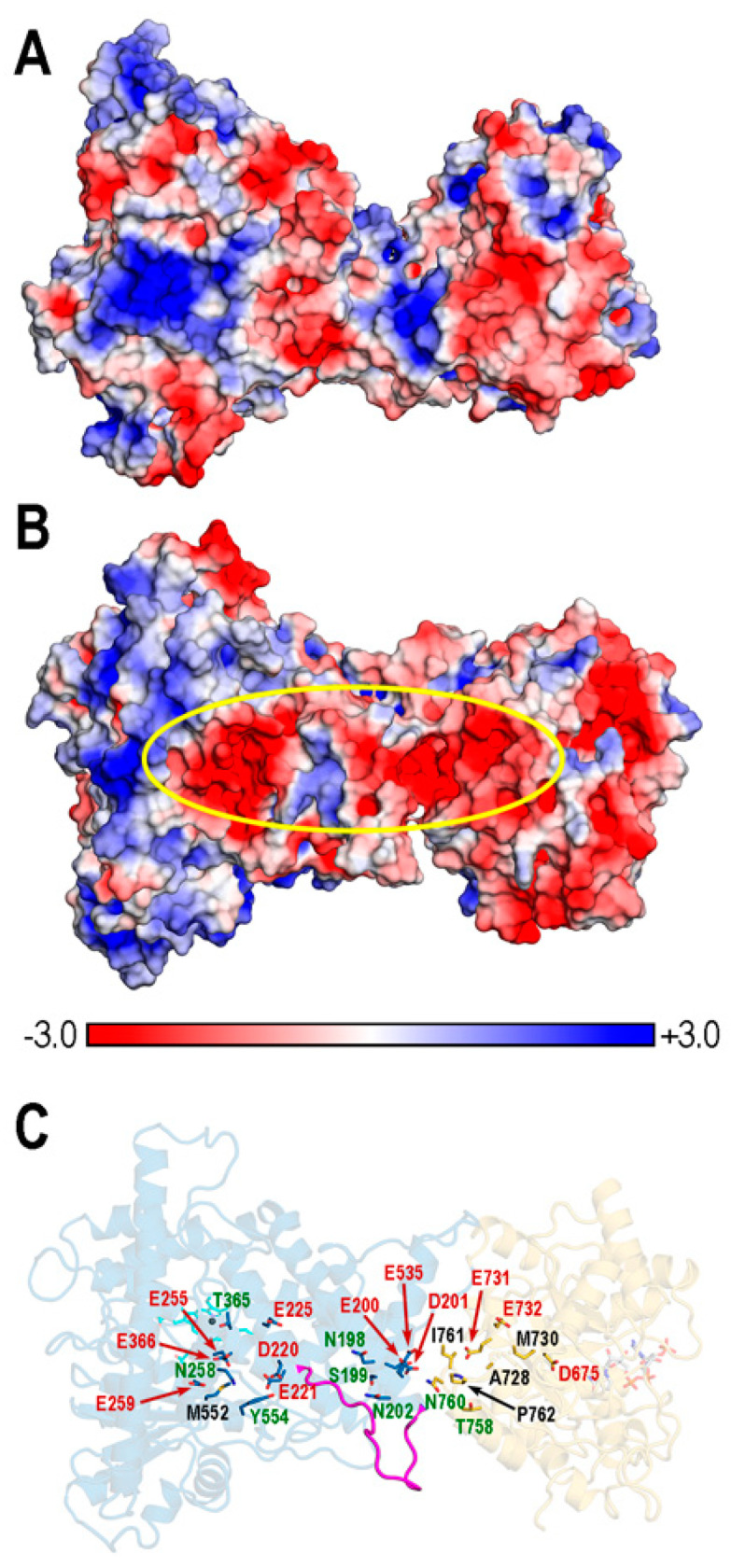
PB-EP mapped onto the protein surface of the final structure after 400 ns MD simulation of the “DER@heme” system. (**A**) Side with PB-EP positive in the interdomain region. (**B**) Side with PB-EP negative in the interdomain region. The scale bar indicates color-coded PB-EP values in units of (*kT/e*). (**C**) Cartoon showing the final structure at the orientation in (**B**). DRS and DRR domains in blue and yellow, respectively. The DRS-DE loop is colored magenta. The large surface patch with negative PB-EP connecting DRS and DRR domains is formed by the surface contributions of residues labeled with colors indicating the amino acid type as follows: red—acidic, negatively charged; green—polar with electronegative atoms; black—nonpolar.

In the exploration of possible tunnels in bifunctional REPI, we found no direct evidence of a tunnel that connects the heme and NADP sites for channeling the DER intermediate. Although our MD results leave open the possibility of tunnel formation upon a conformational change involving the DE loop in the DRS-DRR interdomain (Figure 9), we propose here an electrostatic channeling alternative provided by the analysis of the PB-EP (Figure 10, Figures S11 and S12).

Recalling that the DER intermediate is positively charged, the surfaces of the initial and final structures after MD simulations in the “No ligand” and “REN@heme” systems show no evidence of a negatively charged electrostatic pathway connecting unambiguously the two active sites in REPI (Figure S11). The final structure of “DER@NADPH” exhibits two large negative areas near the interdomain region that even cover both active sites, but they are separated by a neutral–positive patch (Figure S11). Note that in these three systems, the dynamics move the DRS and DRR modules closer together than they are at the start of the simulation, thus increasing the interdomain surface area. The surface opposite to that shown in the three systems of this figure has a rather different electrostatic pattern in the interdomain region, with a large positive area between two domains. In the case of “DER@heme”, however, slightly different electrostatic patterns are observed. Figure S12 displays the PB-EP at four snapshots of the MD simulation, with the final structure (*t* = 400 ns) shown in Figure 10. Note that when the DRS DE loop adopts a closed conformation (*t* = 200 ns), the interdomain surface is greater than in its open conformation (*t* = 120 and 240 ns). Figure 10A represents the opposite surface to that discussed in the other system to illustrate the large positive surface patch in the interdomain region. In contrast, the side shown in Figure 10B has a surface with predominantly negative PB-EP between the two modules that complements the negative regions in DRS and DRR. It is thus tempting to speculate that the surface region marked with the yellow ellipse in Figure 10B might be a possible candidate for an electrostatic highway for channeling DER. In this regard, the nature of the 25 amino acid residues whose surfaces form the region marked in Figure 10B is illustrative: 12 are negatively charged, 8 are polar with electronegative oxygen atoms, and only 5 are non-polar (Figure 10C).

In connection with the speculation in Section 3.6 concerning possible tunnels in REPI, the question of whether shielding 1,2-dehydroreticuline from water could be achieved through electrostatic channeling is a possibility that our MD study also leaves open. The above-mentioned further computational analyses incorporating quantum calculations to address electron and proton transfers at the heme site and the concomitant conformational changes in the DRS module might well reveal an electrostatic channeling of DER as more favorable than tunneling transport. In such a case, the electrostatic highway suggested by our PB-EP results discussed above appears as a strong candidate.

## 4. Conclusions

Studying the natural synthesis of opioids like thebaine, codeine, and morphine in *Papaver somniferum* is crucial for exploring new options in drug synthesis. Specifically, REPI is a key protein in BIA metabolism as it catalyzes the gateway step that leads to morphinan alkaloid biosynthesis. In this study, we used the AlphaFold model of REPI as a basis to first construct its complexes with cofactors (heme and NADP(+)/NADPH) and ligands (REN and DER) and then study their dynamics with all-atom MD simulations. Although the static model structure placed the DRS and DRR domains closely together, the dynamics of all systems (apo state and three complexes) revealed that both modules move with respect to one another thanks to the flexibility of a 10-residue segment that links them. The separate internal mobility of DRS and DRR is far smaller than that of the whole REPI protein, a result consistent with the great stability of their architectures: the cytochrome P450 in DRS and the TIM-barrel characteristic of AKRs in DRR. The dynamics of REPI complexes were dependent on whether the ligand is REN (substrate of DRS) or DER (product of DRS and substrate of DRR). A REPI complex with DER located at the heme site in DRS was included to explore the dynamics of a system in which DER should migrate to DRR. Systems with no ligand or with DER at DRS showed large relative motions of the DRS and DRR modules, yielding final structures after an MD simulation with rather different spatial positions to those of the initial structures. In contrast, the complexes with the ligands on their corresponding binding sites exhibit consistently short distances over the full simulation, thus suggesting that the presence of a ligand causes the DRS and DRR modules to move closer to each other.

The results in the present study provide additional insight into the activity of the AKR module in REPI (DRR), as compared with the closely related COR protein. The dynamics of the complex with DER at DRR confirms the previously reported proposals [19] about the different catalytic activities of DRR and COR as the catalytic tetrad typical of AKRs is not conserved in DRR. However, our MD data unveiled the presence of a lysine residue (K607) not present in COR that could play a relevant role in the reductase function of DRR. Our results also confirm the different nature of the substrate binding site in REPI-DRR with respect to COR, although two residues in DRR relevant for binding DER (F700 and F882) are spatially coincident with two equivalent residues in the binding site of COR (H120 and F302).

Furthermore, our MD study unveiled the key roles played by some secondary structure elements of DRS in the dynamics of REPI complexes, namely the D and J helices and DE and Jg2 loops. The DE loop happens to play a particularly relevant role in the complex with DER at the heme site (DRS), changing its geometry from an open conformation to a closed conformation at time intervals. The closed conformation of the DE loop acts as a wall in the interdomain space, favoring, in turn, the transient occurrence of possible tunnels that would be associated with the internal channeling of the intermediate from DRS to DRR. In this same complex, a large surface region with negative electrostatic potential connecting both domains that would act as a pathway for the surface electrostatic channeling of the intermediate was also found. Although these results provide structurally insightful information about the bimodular function of the REPI protein, further quantum calculations and metadynamics simulations should be built upon the structural characterization presented here.

This work has contributed contributes to a deeper understanding of REPI structure, encompassing substrate binding sites, and molecular dynamics. This knowledge is crucial for enhancing the catalytic activity of BIA enzymes when attempting to replicate the biosynthesis of these plant-derived therapeutic molecules in heterologous expression systems. Notably, protein engineering strategies have emerged as powerful tools for modifying the substrate specificity of known enzymes to stablish novel metabolic pathways [88]. Our study will significantly contribute to the efficiency of microbial BIA biosynthesis, improving the supply chain of these valuable drugs.

## Figures and Tables

**Table 1:** Average and standard deviation values of total non-bonded (electrostatic + VdW terms) interaction energies between DRS and heme cofactor (“DRS-heme”), between DRR and NADP(+) or NADPH cofactor (“DRR-NADP”), and between DRS/heme and REN or DER, and between DRR/NADPH and DER (“Enzyme-substrate”) computed along the MD simulations for the four REPI systems studied (all values in kcal/mol).

	DRS-Heme		DRR-NADP		Enzyme-Substrate	
Complex	Average	Std. Dev.	Average	Std. Dev.	Average	Std. Dev.
No ligand	−130.7	4.1	−153.1	6.0	-	-
REN@heme	−134.2	5.3	−154.4	15.3	−40.1	3.0
DER@heme	−132.9	7.4	−159.9	13.2	−54.3	3.9
DER@NADPH	−106.4	17.2	−155.2	7.7	−37.0	4.3

**Table 2:** Top: average and standard deviation values of the number of hydrogen bonds (#HBs) between cofactors (heme and NADP(+)/NADPH) and water and between ligands (REN and DER) and water. Bottom: average and standard deviation values of the number water molecules (#waters) in a neighborhood of 5 Å around any cofactor atom (heme and NADP(+)/NADPH).

	#HBs Heme–Water		#HBs NADP–Water		#HBs Ligand–Water	
Complex	Average	Std. Dev.	Average	Std. Dev.	Average	Std. Dev.
No ligand	3.2	0.5	5.7	1.0	-	-
REN@heme	2.3	0.4	6.3	0.9	1.0	0.2
DER@heme	2.7	0.9	5.6	1.7	0.5	0.3
DER@NADPH	4.5	1.5	7.1	0.8	0.7	0.3
	**#waters-heme**		**#waters-NADP**			
No ligand	19.2	3.0	46.1	8.0		
REN@heme	24.1	4.9	50.4	5.5		
DER@heme	15.8	3.3	42.9	6.0		
DER@NADPH	36.6	7.7	46.2	5.1		

## Data Availability

The data presented in this study are contained within the article.

## Supplementary Materials

The following supporting information can be downloaded at https://www.mdpi.com/article/10.3390/biom14010002/s1. Table S1: Secondary structure assigned to DRS and DRR modules of REPI. Figure S1: AlphaFold Database model structure of REPI; Figure S2: Model structures of REPI obtained with AlphaFold2 software; Figure S3: variation over the 400 ns MD simulations in secondary structure of DRS domain; Figure S4: variation over the 400 ns MD simulations in secondary structure of DRR domain; Figure S5: FG loop of DRS domains and comparison of ligand geometries in crystal structures of plant P450 cytochromes and in model structures of REPI-DRS domains; Figure S6: four snapshots of the MD simulation for “DER@heme” complex; Figure S7: variation over the 400 ns MD simulations in the number of hydrogen bonds between water and cofactors or ligands; Figure S8: variation along the 400 ns MD simulations in the number of water molecules at 5 Å from heme and NADP(+) or NADPH cofactors; Figure S9: variation along the 400 ns MD simulations in non-bonded (electrostatic + VdW) energies for the DRS-heme interaction in the “DER@NADPH” complex; Figure S10: Tunnels found with MOLE 2.5 in the final structures of “No ligand”, “REN@heme”, and “DER@NADPH” systems; Figure S11: initial and final structures and PB-EP mapped onto their surfaces in the “No ligand”, “REN@heme”, and “DER@NADPH” systems; Figure S12: Structures and PB-EP mapped onto their surfaces corresponding to four snapshots of the MD simulation of “DER@heme” system.
