# Supplementary material for: Structural Characterization and Molecular Dynamics Study of the REPI Fusion Protein from Papaver somniferum L
Source: Biomolecules. 2023 Dec 19;14(1):2. doi: 10.3390/biom14010002 (PMC10813097; doi:10.3390/biom14010002)
Supplement: Supplementary file 1 [file biomolecules-14-00002-s001.zip › biomolecules-2756801-supplementary.pdf]

Structural characterization and molecular dynamics study of the REPI fusion protein from *Papaver Somniferum* L.

Alba Diaz-Bárcena*, Luis F. Pacios, Patricia Giraldo

Supplementary Materials

	Page
**Table S1**	**2**
**Figure S1**	**3**
**Figure S2**	**4**
**Figure S3**	**5**
**Figure S4**	**5**
**Figure S5**	**6**
**Figure S6**	**7**
**Figure S7**	**8**
**Figure S8**	**9**
**Figure S9**	**9**
**Figure S10**	**10**
**Figure S11**	**11**
**Figure S12**	**12**

Table S1. Assignment of secondary structure in the AlphaFold model structure of REPI. The labeling is that conventionally used in the literature [39,69] for the DRS domain (residues 46-570) and that proposed in the study on COR protein [19] for the DRR domain (residues 571-901).

DRS			DRR		
α–helix	*β–strand*	*3_{10} helix*	*α–helix*	*β–strand*	*3_{10} helix*
A′ 52-58	β1-1 93-98	g1 68-72 (2)	α1 613-625	β1 596-602	γ1 581-584 (5)
A 79-90	β1-2 101-106	g2 414-419 (1)	α2 639-652	β2 629-631	γ2 634-636 (5)
B 109-115	β1-3 460-463	g3 483-487 (1)	α3 677-688	β3 662-666	γ3 658-660 (5)
α1 118-123 (1)	β1-4 440-443		α4 723-735	β4 693-698	γ4 715-717 (5)
B′ 131-136	β2-1 448-450		α5 749-755	β5 739-745	γ5 881-883 (5)
C 155-167	β2-2 101-106		α6 777-785	β6 765-769	
C′ 171-176	β3-1 216-218		α7 825-835	β7 789-793	
D 178-201	β3-2 560-565		α8 847-854	β8 839-842	
E 219-236	β3-3 534-537 (1)		H1 812-821	b1 588-590 (3)	
F 249-265			H2 863-869	b2 702-704 (4)	
α2 278-282 (1)			a1 893-896 (3)	b3 718-720 (4)	
G 285-314					
H 329-337				Loop A 705-714	
I 351-380				Loop B 794-811	
J 383-404				Loop C 871-892	
J′ 408-410				Loop β1α1 603-612	
K 421-433				Loop β2α2 632-638	
K′ 465-468					
α3 489-492 (1)					
L 516-533					

(1) Assigned coil in [19,68]. (2) Labelled as α-helix "a" in [39,69]. (3) α-helix or β-strand with no symbol in [19]. (4) β-strand absent in [19]. (5) 3_{10} helices not assigned in [19].

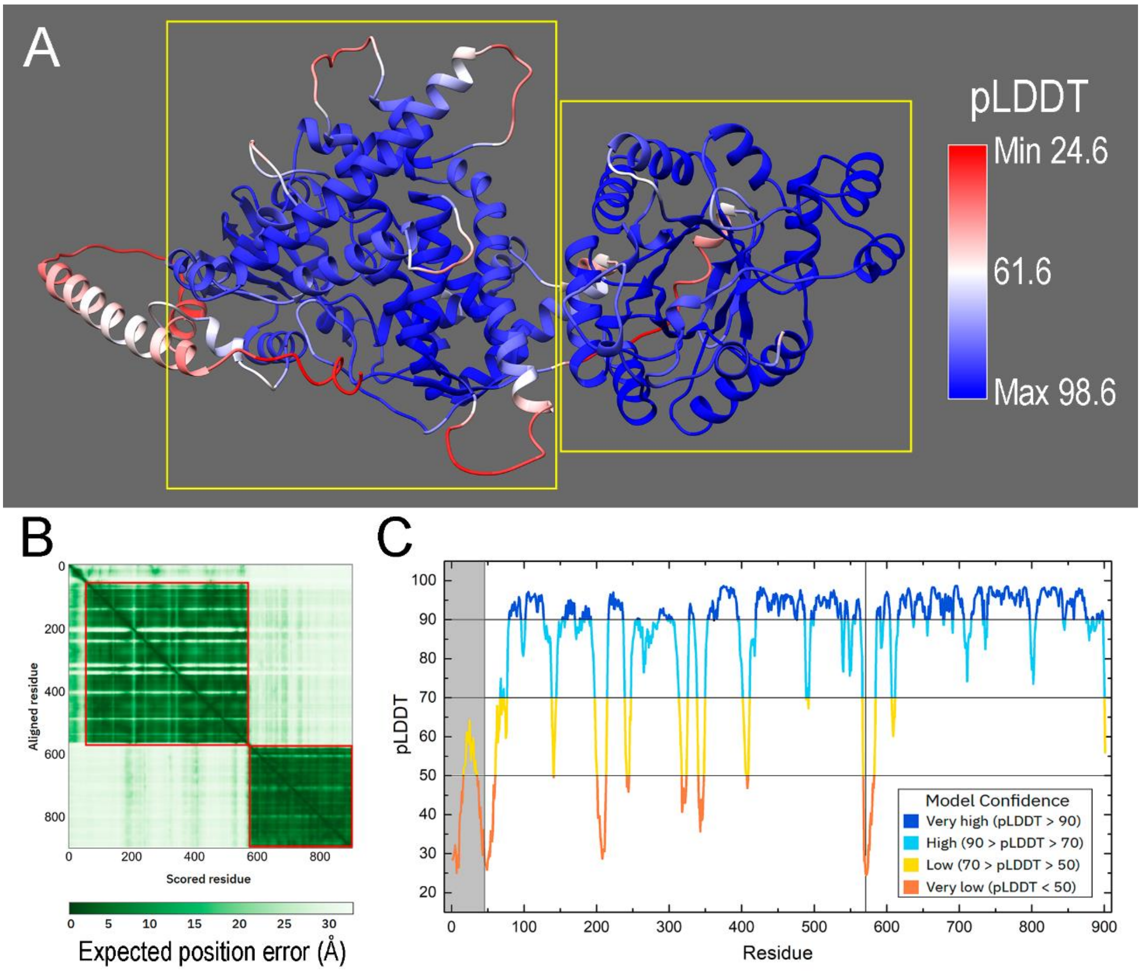

Figure S1. AlphaFold model structure of REPI in the AlphaFold Protein Structure Database. **(A)** Model colored in a red-white-blue palette for the predicted Local Distance Difference Test (pLDDT) scale indicated on the right. Yellow boxes enclose DRS domain (left) spanning residues 46-570 and DRR domain (right) spanning residues 571-901. The segment outside DRS box spans residues 1-45 and includes an N-terminal disordered region 1-13, transmembrane α-helix 14-39, and a short loop 40-45. This 1-45 segment was removed from the REPI structure studied here. **(B)** Predicted alignment error (PAE) giving the expected distance error (Å) in a residue at X when the predicted and true structures are aligned on a residue at Y. PAE map serves to define different domains, here DRS (left red box) and DRR (right red box) marked in **A**. (**C**) Plot of per-residue pLDDT metrics measuring the confidence of the local structure for each amino acid on a scale 0-100 colored according to the AlphaFold prescription in the label. The shaded area marks the 1-45 segment discarded in our study and the vertical line at residue 571 separates DRS and DRR domains which are linked by the unstructured segment 568-577.

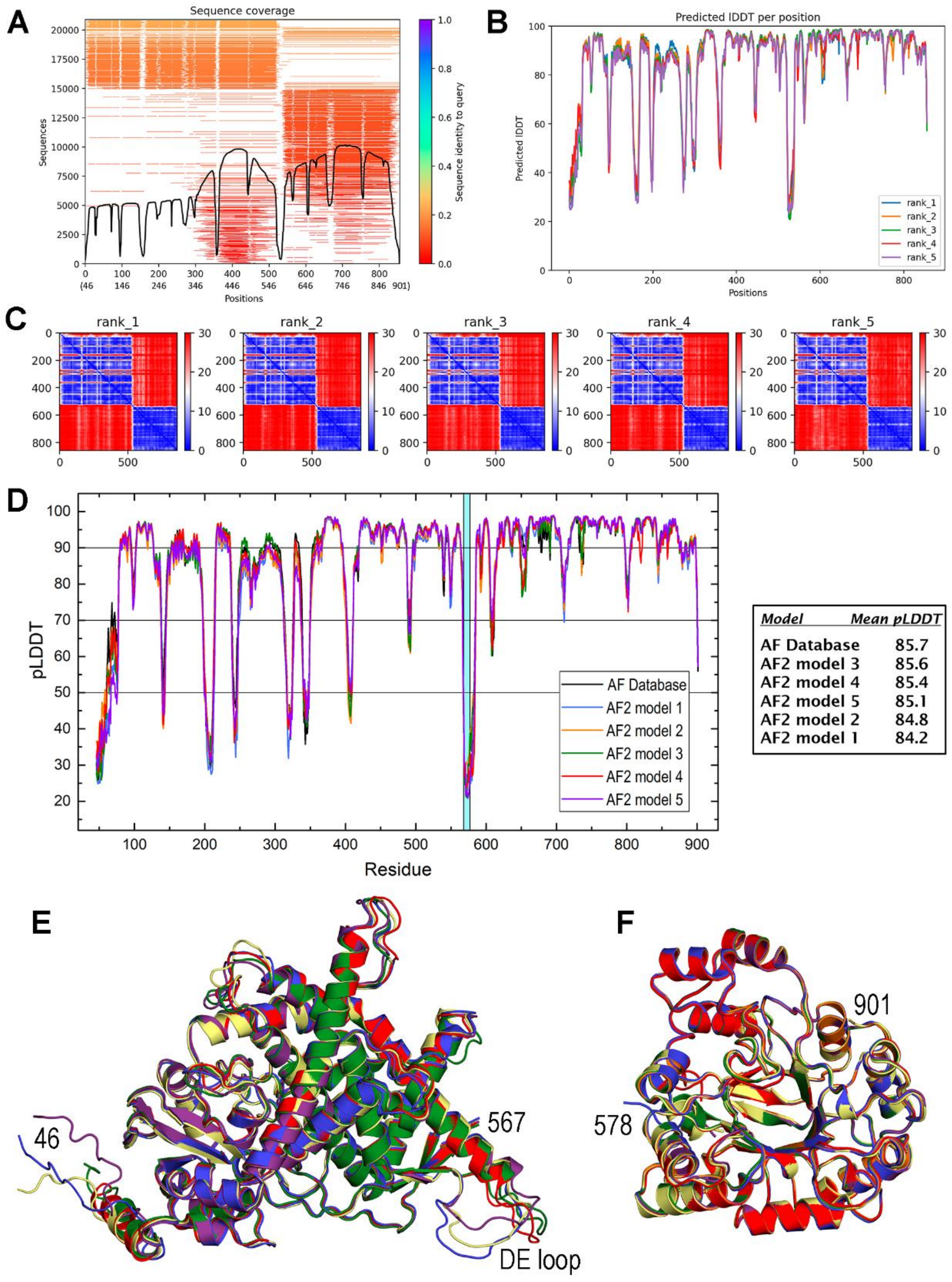

Figure S2. Structure of REPI (residues 46-901) modeled with AlphaFold2 (AF2) software. **(A)** Sequence coverage. (**B**) Per-residue pLDDT and (**C**) PAE maps for the five models generated with AF2. Plots **A**-**C** are provided in the AF2 standard output. (**D**) Per-residue pLDDTs for the five AF2 models (AF2 colors) and the model downloaded from the AlphaFold Database (AFDB, black). Area shaded blue corresponds to the interdomain linking segment 568-577. Horizontal lines separate the pLDDT confidence ranges indicated in Figure S1C. The table on the right gives the mean pLDDT values for the six models ranked from highest to lowest confidence. (**E**) Superposition of DRS domains (46-567) in the five AF2 models (same colors as in **B** and **D**) and the AFDB model (light yellow). (**F**) Same as **E** for DRR domains (578-901).

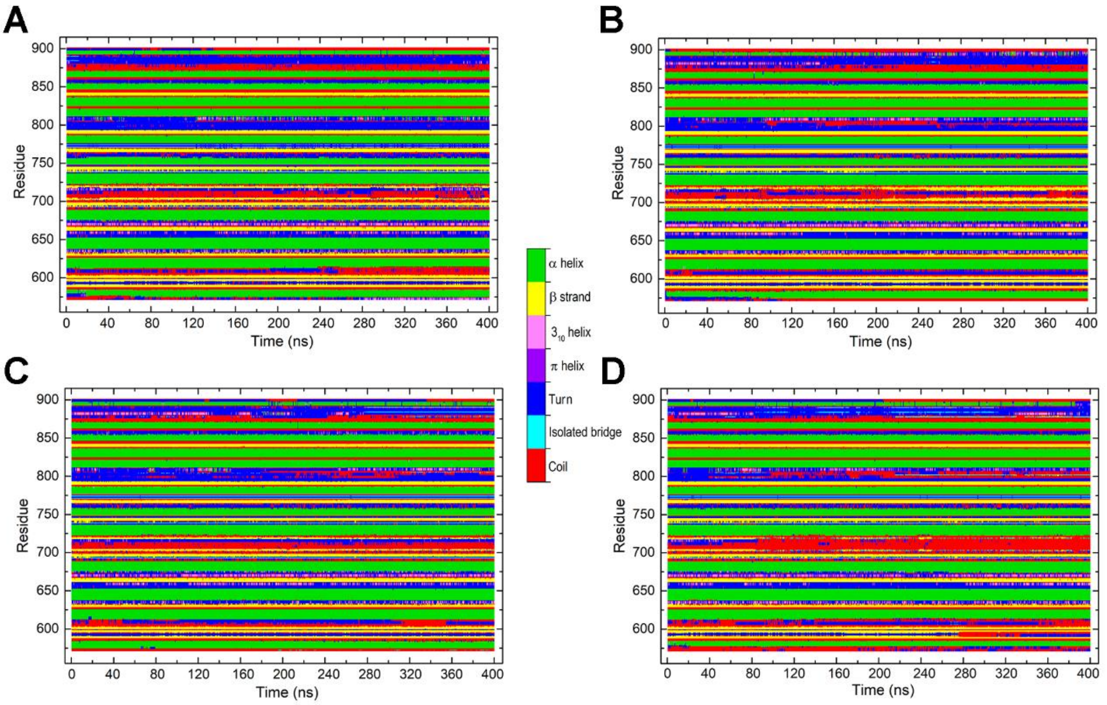

Figure S3. Variation along the 400-ns MD simulations of secondary structure for the DRS domain in the four REPI complexes studied: (**A**) “No ligand”, (**B**) “REN@heme”, (**C**) “DER@heme”, and (**D**) “DER@NADPH”.

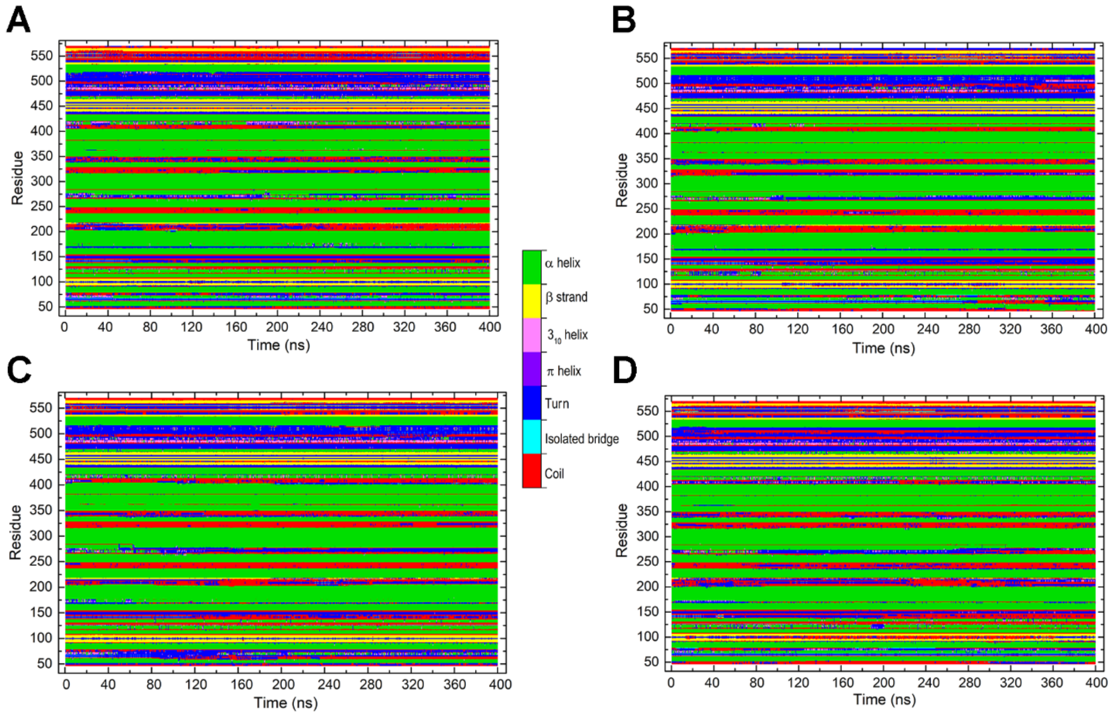

Figure S4. Variation along the 400-ns MD simulations of secondary structure for the DRR domain in the four REPI complexes studied: (**A**) “No ligand”, (**B**) “REN@heme”, (**C**) “DER@heme”, and (**D**) “DER@NADPH”.

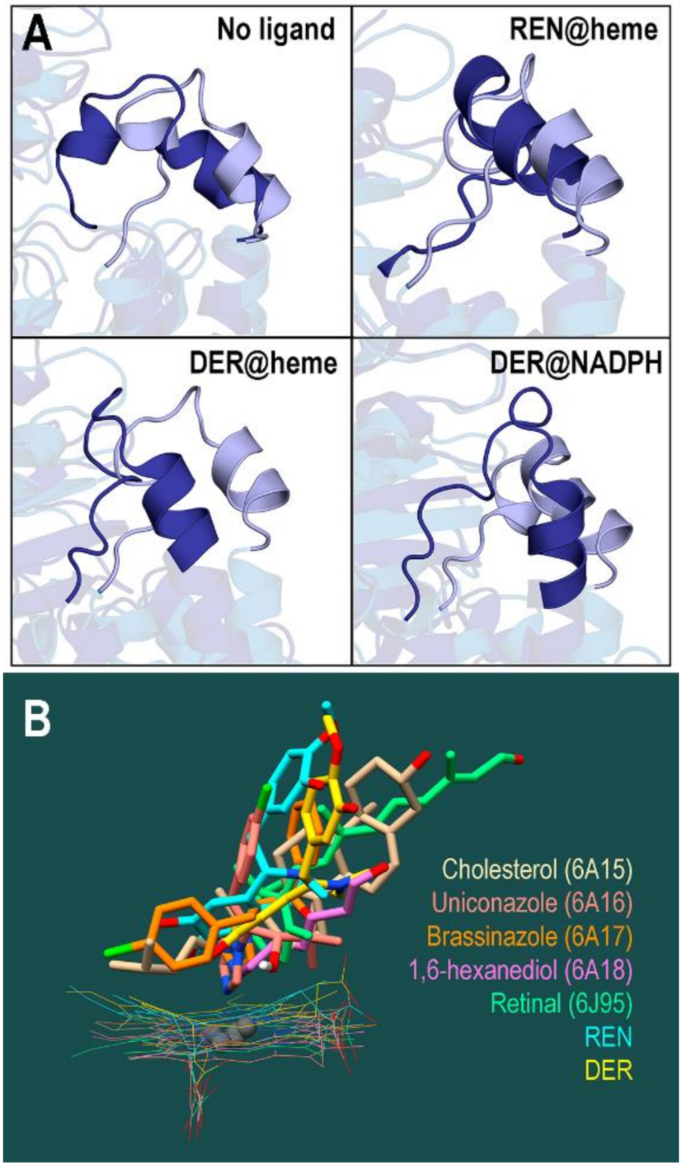

Figure S5. (A) FG loop (residues 266-284 including the α2 helix 278-282) of the DRS domain in the superposition of initial (light blue hues) and final (deep blue hues) structures after the 400-ns MD simulations. (**B**) Comparison of the geometries of different substrates (sticks) at heme (lines and grey spheres for iron) site in crystal structures (PDB ids. in parentheses; 6A15, 6A16, 6A17, and 6A18: [73]; 6J95: [83]) and in the initial model structures of "REN@heme" and "DER@heme" complexes.

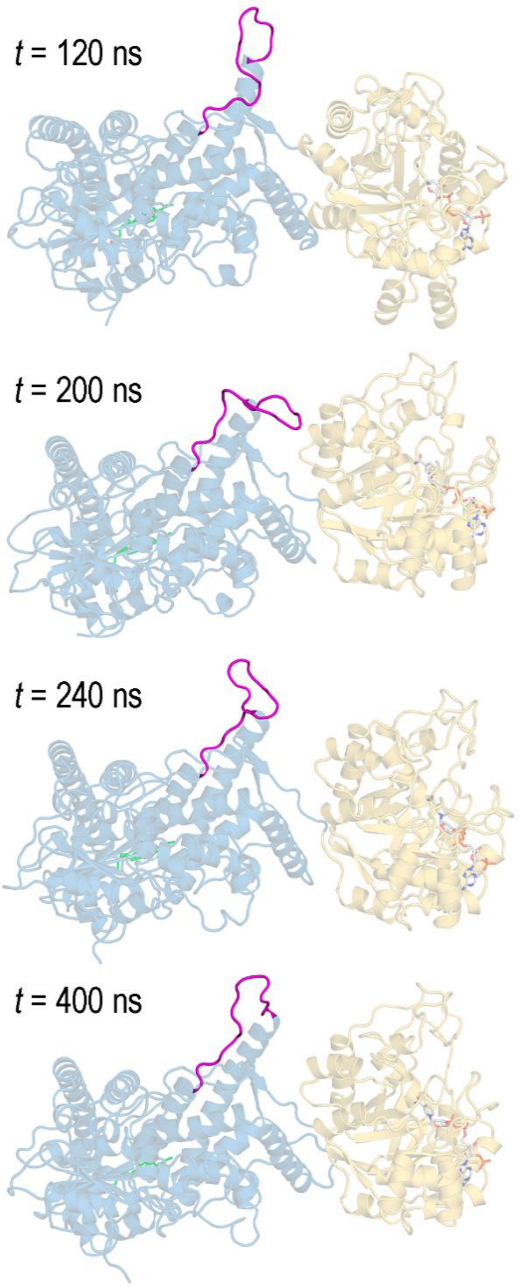

Figure S6. Four snapshots at the indicated times of the MD simulation for the "DER@heme" system. DRS and DRR domains are shown as 70% transparent cartoons in blue and yellow, respectively. The DE loop of DRS is highlighted in magenta at no transparency.

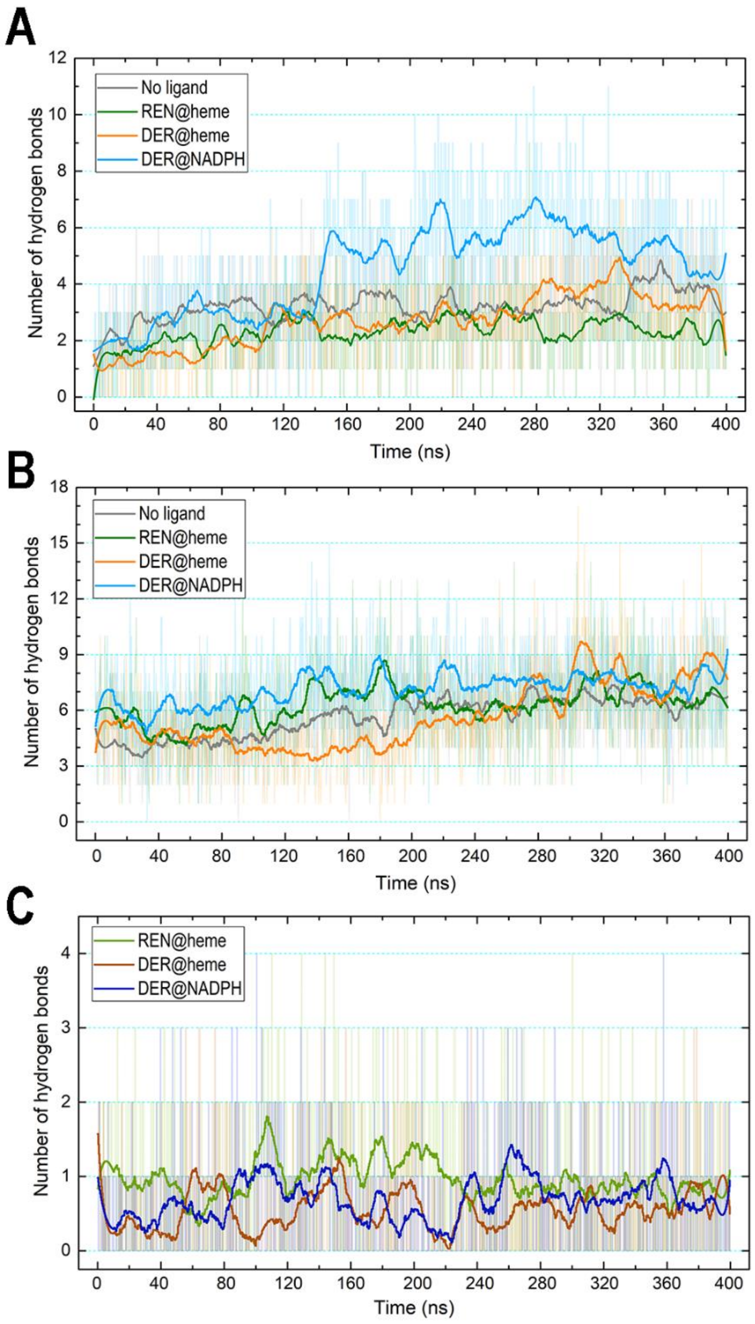

Figure S7. Variation along the MD simulations of the number of hydrogen bonds (HBs) between cofactors or ligands and water molecules. (**A**) HBs heme - water. (**B**) HBs NADP(+) or NADPH - water. (**C**) HBs ligands (REN or DER) - water. Solid curves correspond to 51-points 3rd order Savitzsky-Golay polynomial smoothing of discrete data shown in 80% transparency.

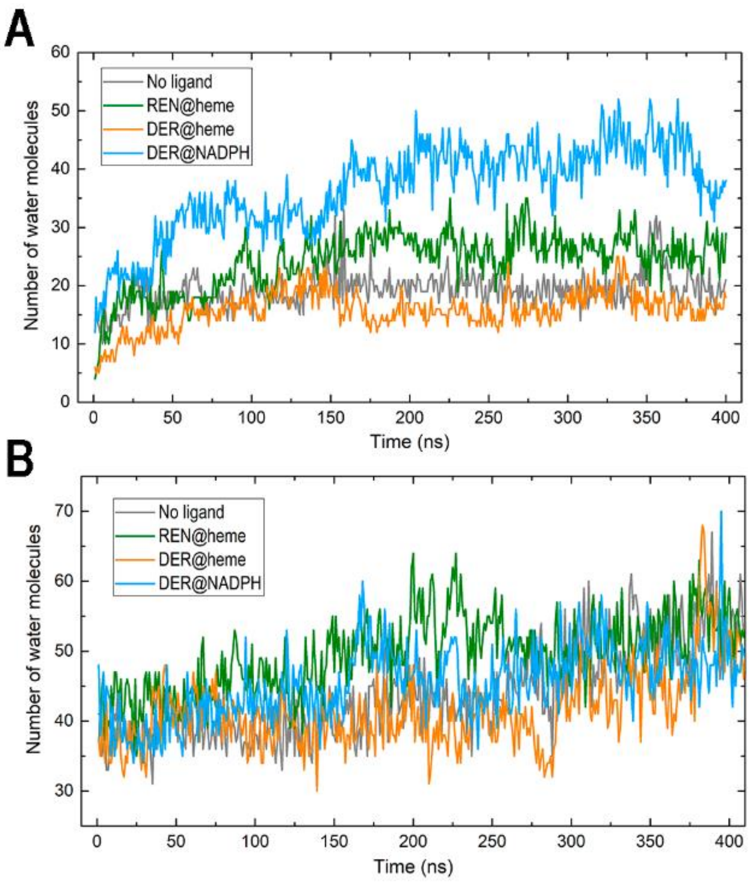

Figure S8. Variation along the 400-ns MD simulations of the number of water molecules at 5 Å from any atom of (**A**) heme and (**B**) NADP(+) or NADPH cofactors.

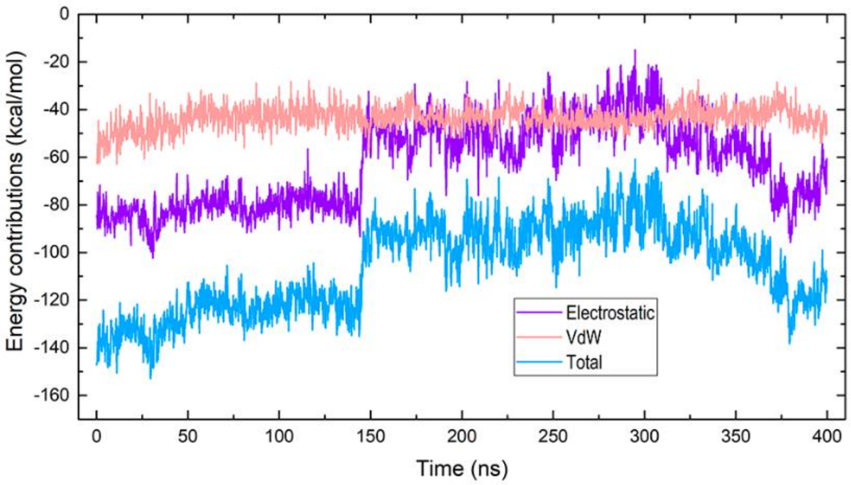

Figure S9. Variation along the 400-ns MD simulations of non-bonded energies for the interaction between DRS and heme cofactor in "DER@NADPH" complex. Total energy = electrostatic + VdW terms.

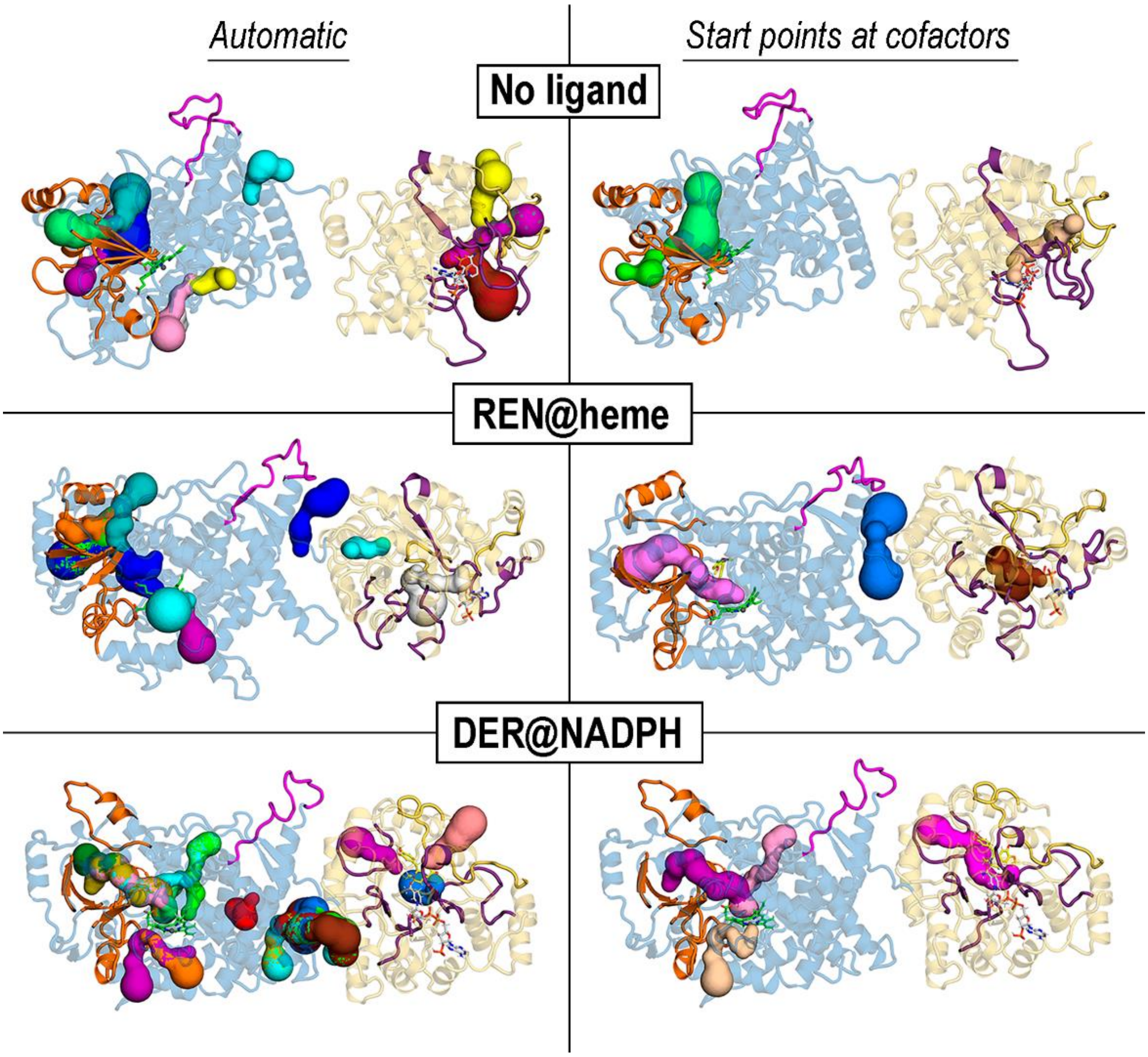

Figure S10. Tunnels found with MOLE 2.5 using the *automatic* selection of start points (left column) and user-defined *start points at cofactors* heme and NADP(+)/NADPH (right column) in the final structures after 400-ns MD simulations of "No ligand", "REN@heme", and "DER@NADPH" complexes. Same graphical options as used in Figure 9. In *Start points at cofactors* column, tunnels lying in DRS were found when only heme was selected as start point, those lying in DRR were found when only NADP(+)/NADPH was selected as start point, and the single tunnel in the interdomain space in "REN@heme" was found when both cofactors were together selected as start points. This latter selection yielded no interdomain tunnels in "No ligand" and "DER@NADPH" systems.

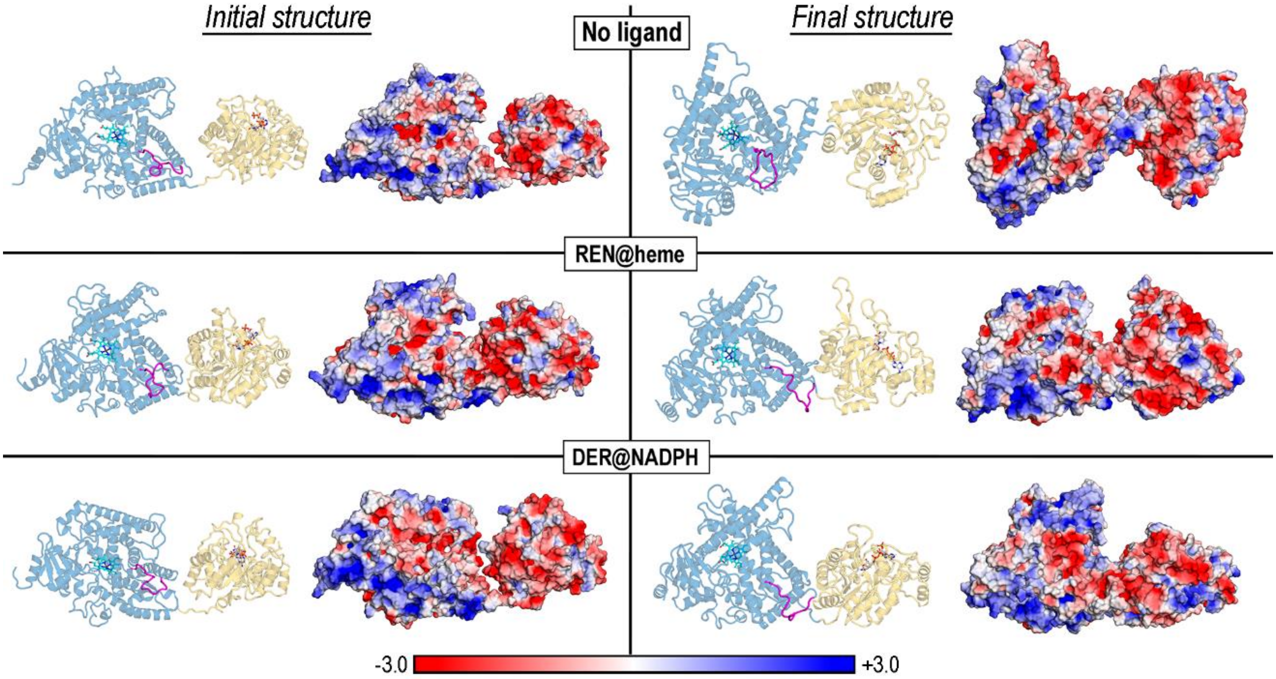

Figure S11. Initial and final structures and PB-EP mapped onto their molecular surface in MD simulations of "No ligand", "REN@heme", and "DER@NADPH" systems. DRS and DRR domains in blue and yellow, respectively. DRS-DE loop is colored magenta. The scale bar indicates color-coded PB-EP values in units of (*kT*/*e*).

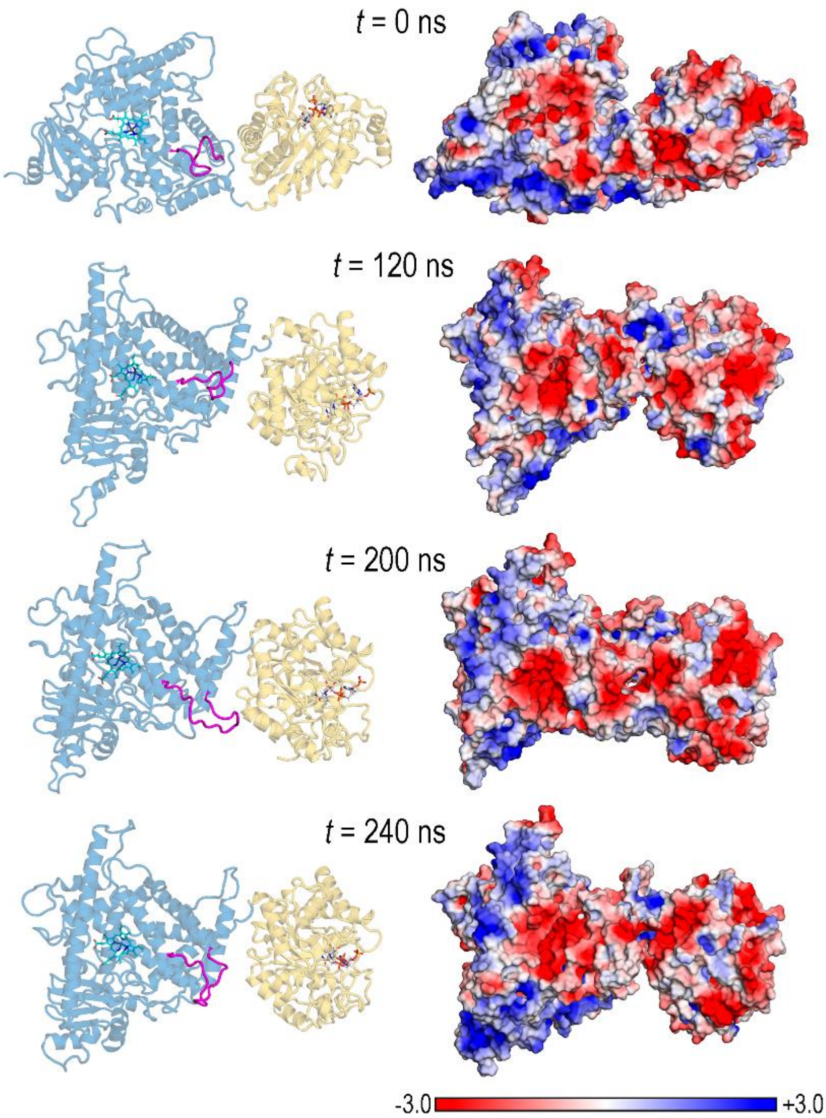

Figure S12. Structures and PB-EP mapped onto their molecular surface corresponding to four snapshots at the indicated times of the MD simulation for "DER@heme" system. DRS and DRR domains in blue and yellow, respectively. DRS-DE loop is colored magenta. The scale bar indicates color-coded PB-EP values in units of (*kT*/*e*).
